# Assessing Insect Growth Regulator Resistance Using Bioassays: A Systematic Review and Meta-Analysis of Methoprene and Pyriproxyfen Inhibition of Emergence in Three Vector Mosquito Species

**DOI:** 10.3390/tropicalmed10040087

**Published:** 2025-03-28

**Authors:** Mark E. Clifton, Kristina Lopez

**Affiliations:** North Shore Mosquito Abatement District, 117 Northfield Rd, Northfield, IL 60093, USA

**Keywords:** *Aedes*, *Culex*, insecticide resistance, larvicide, susceptibility

## Abstract

This systematic review and meta-analysis aims to: (1) characterize the distribution of published inhibition of emergence (IE_50_, IE_90_, and IE_95_) reference values for pyriproxyfen and methoprene in *Culex pipiens* [L.], *Aedes aegypti* [L.], and *Aedes albopictus* [Skuse]; (2) generate combined-effect IE values using a DerSimonian and Laird (DL) random-effects model to establish benchmarks for future resistance assessments; and (3) compare these combined-effect IE values with previously published literature. A systematic search was conducted in PubMed, SciELO, J-STAGE, and Google Scholar up to 10 February 2025, following the Preferred Reporting Items for Systematic Reviews and Meta-Analyses (PRISMA) guidelines. Eligible studies were primary, peer-reviewed literature that aligned with World Health Organization (WHO) protocols for insect growth regulator (IGR) resistance testing, specifically those reporting susceptible reference IE values from continuous immersion dose-response bioassays analyzed using probit regression. A total of 72 unique studies that aligned with WHO protocols were assessed for publication bias using a funnel plot and Egger’s regression. Sensitivity and subgroup analyses were conducted to evaluate individual study contributions to the overall combined effect. Heterogeneity (I^2^) and combined effect values were estimated for 18 different species/active ingredient/IE concentration subgroup pairings. Heterogeneity (I^2^) ranged from 29.32 to 99.78% between the 18 subgroups, indicating inconsistency within the literature. The DL combined effect IE_50_ varied from 0.048 ppb for *Cx. pipiens* exposed to pyriproxyfen to 1.818 ppb for *Ae. albopictus* exposed to methoprene. A certainty analysis indicated that 1 combined effect value exhibited high certainty, 8 out of 18 pairings were moderately certain, 6 exhibited low certainty and 3 exhibited very low certainty. The main causes of uncertainty (ranked) were inconsistency between studies, imprecision of the combined effect size, and possible publication bias. Our findings indicate that (1) robust DL combined effect IE_50_ values could be established for all species/IGR pairings, providing essential benchmarks for future resistance assessments; (2) substantial heterogeneity among susceptible laboratory colonies complicates resistance detection in field-collected mosquitoes; and (3) a significant portion of the literature relies on reference mosquito strains that are likely not fully susceptible, further complicating resistance detection. This study was not registered and was supported by the North Shore Mosquito Abatement District.

## 1. Introduction

Mosquito-borne diseases remain a global public health burden, with increasing mortality and morbidity rates anticipated because of globalization and climate change [1,2,3]. The mosquitoes, *Culex pipiens* [L.], *Aedes aegypti* [L.], and *Aedes albopictus* [Skuse] are globally invasive vector mosquitoes which together have been implicated in various outbreaks of Zika, chikungunya, West Nile virus, and dengue fever [1,2]. Some notable examples of outbreaks caused by these mosquitoes include 1868 cases and 89 deaths attributed to West Nile virus in Dallas, Texas, in 2012, vectored by *Cx. pipiens quinquefasciatus* [Say], over 42,000 locally acquired cases of Zika in Puerto Rico and the US territories in 2016 and 2017 vectored by *Ae. aegypti*, and a 2005 outbreak of chikungunya vectored by *Ae. albopictus* on the island of Réunion which saw over 250,000 cases [1,2]. Due to the absence of widely available preventive vaccines or prophylactic treatments, mosquito population abundance reduction remains a central strategy for many vector control programs. For over a century, mosquitoes have faced various selective pressures from chemical interventions aimed at reducing populations and mitigating the risk of disease transmission [4,5]. Larval mosquito control has long been recognized as a vital element in any strategy for population management and diverse classes of larvicides have been developed to support this objective [6]. Not surprisingly, resistance to nearly every class of larvicide has been observed around the world both in wild populations as well as laboratory-selected colonies [7,8].

Insect growth regulators (IGRs), such as methoprene and pyriproxyfen, were first developed and introduced in the 1970s as “third-generation” pesticides [9,10,11]. These pesticides were designed to offer greater selectivity, fewer non-target effects, and a reduced likelihood of resistance development [7,12,13,14]. By interfering with gene expression during metamorphosis, the juvenoid IGRs can prevent the development and successful emergence of adult mosquitoes [15,16]. Despite the reduced potential for resistance, a variety of reports over recent decades provide overwhelming evidence that, even for juvenoid IGRs, the development of resistance is possible in wild mosquito populations [7,8].

To maintain an arsenal of effective larval control materials, it is necessary for public health and other vector control programs to periodically assess wild populations of mosquitoes for susceptibility to existing or potential control materials. The most widely utilized methodology for assessing IGR susceptibility requires a phenotypic bioassay methodology where susceptible larval mosquitoes (most commonly a laboratory-maintained “reference” colony) and field-collected mosquito populations are exposed to a range of concentrations of the IGR in parallel with untreated controls [17,18]. Corrected mortality at each concentration is subjected to a probit regression analysis to determine the concentration of IGR that inhibits emergence (IE) of 50, 90, 95%, etc., of larval mosquitoes (i.e., IE_50_, IE_90_ and/or IE_95_).

The baseline “reference” value derived from this procedure can then be used to develop a “discriminating dose” or “diagnostic dose”. For example, the World Health Organization (WHO) recommends two times the IE_99_._9_ (the concentration that causes inhibition of emergence in 99.9% of exposed larvae) of a susceptible reference strain as a diagnostic dose [17]. The diagnostic dose can then be applied to field-collected mosquitoes to determine susceptibility as compared to the reference strain. This method reduces the dependence on a reference colony, which is especially important in geographies or for species where a reference colony may be unavailable. Alternatively, a ratio can be calculated which, most commonly, compares the IE_50_ value of field-collected mosquitoes to that of the susceptible reference strain to create a resistance ratio or “RR” [19]. The WHO has identified resistance ratios at the IE_50_ of <5 as indicating susceptible field populations; an RR between 5 and 10 indicates moderate resistance, and an RR > 10 indicates high resistance [18]. For both the diagnostic dose method and the resistance ratio method, the susceptible reference IE value serves as the only baseline for comparison and so the reliability of this value is critically important for understanding the presence and intensity of resistance that may exist.

WHO standard assessments of mosquito susceptibility to IGR larvicides rely entirely on mathematical comparisons to a baseline susceptible reference IE value. As a result, variability in reference measurements—whether due to bias, experimental errors, or biological differences between reference colonies—can obscure resistance detection or lead to inaccurate assessments of its intensity. For instance, if a susceptible reference colony has an IE_50_ value that is too high, the resulting RR calculation will be artificially low. The relationship between the reference IE values and the resulting RR follows a rectangular hyperbola, where small changes in the denominator (susceptible IE values) can cause disproportionately large shifts in RR.

Any resistance assessment based on an inaccurate reference baseline risks leading to inappropriate treatment decisions, reduced field effectiveness, or even treatment failures. Although the WHO has published protocols intended to standardize the experimental conditions for deriving reference IE values, the extremely wide variation in published reference IE values significantly limits the interpretation of resistance assessments and complicates comparisons between studies and regions. In addition, the WHO does not report pre-established standard IE values for susceptible mosquito populations. This necessitates individual research groups acquire susceptible strains, maintain said mosquito strain in colony, and evaluate for insecticide susceptibility. The lack of comparability and reliability in susceptible reference measurements has important ramifications for public health and mitigating the threat posed by mosquito vectors of disease. Therefore, understanding the range and variability in published reference values is crucial for evaluating the validity and reliability of experimentally derived IE values, as well as for assessing the ongoing susceptibility of reference colonies maintained globally. Given these challenges, our study aims to systematically assess and synthesize reference IE values to improve the reliability of IGR resistance assessments.

The specific goals of this systematic review and meta-analysis are threefold. First, we aim to summarize and characterize the distribution of published susceptible reference IE values (IE_50_, IE_90_, and/or IE_95_) for three globally invasive vector mosquito species (*Cx. pipiens, Ae. aegypti*, and *Ae. albopictus*) and two IGRs (methoprene and pyriproxyfen). Second, we aim to generate composite IE values for each species, IE value, and IGR using a DerSimonian and Laird random-effects model. A robust susceptible reference value, synthesized from multiple studies, can serve as a crucial benchmark for public health and vector control professionals to assess susceptibility in field-collected mosquitoes, evaluate the suitability of mosquitoes for use as susceptible references, and assess or monitor the continued susceptibility of laboratory colonies. These values can be used when access to or maintaining a reference colony is unattainable, a common barrier in the mosquito control field [20], or to validate susceptibility of existing strains. Finally, we aim to compare synthesized IE values with previously published susceptible reference values to contextualize the existing literature and highlight trends or discrepancies in resistance assessment.

## 2. Methods

The Preferred Reporting Items for Systematic Reviews and Meta-Analyses (PRISMA) 2020 standard guidelines were used to address the main goals of this study [21,22]. The PICO framework was used as follows: Population = pesticide-susceptible laboratory colony mosquitoes intended as a susceptible control or reference population. Intervention = exposure to the insect growth regulators methoprene or pyriproxyfen in a bioassay aligned with WHO protocols;. Comparison = variability in baseline susceptibility reported across the literature. Outcome = IE_50_, IE_90_, and/or IE_95_ values derived from a probit-based analysis [23]. This meta-analysis was not prospectively registered, as it did not assess a specific hypothesis, treatment effect, or treatment efficacy but instead aimed to examine the variability in control values from previously published research. Given this study’s focus on methodology rather than hypothesis testing or treatment effect quantification, prospective registration was not deemed necessary.

### 2.1. Literature Search Strategy

The goal of the literature search was to identify reference emergence of inhibition concentrations (IE_50_, IE_90_, and/or IE_95_ values) from three vector species of pesticide-susceptible laboratory mosquitoes exposed to the insect growth regulators methoprene or pyriproxyfen derived using a WHO-aligned dose/concentration-response bioassay. Both authors independently conducted a systematic search of internet databases to identify relevant publication records. Four databases were queried between 3 September 2024 and 10 February 2025: PubMed (last searched 7 February 2025), SciELO (last searched 7 February 2025), J-STAGE (last searched 7 February 2025), and Google Scholar (last searched 10 February 2025). The following keywords and combinations of keywords were used:

PubMed, SciELO, and J-STAGE search terms:

“Aedes aegypti” AND “methoprene” OR “pyriproxyfen” OR “s-31183”

“Aedes albopictus” AND “methoprene” OR “pyriproxyfen” OR “s-31183”

“Culex” AND “methoprene” OR “pyriproxyfen” OR “s-31183”

Google Scholar search terms:

“Aedes aegypti” OR “Aedes albopictus” OR “Culex” AND “Methoprene” OR “pyriproxyfen” OR ““s-31183” AND “emergence inhibition” AND “probit”

There were no search limitations imposed for date or language of publication. All search keywords were evaluated against the full text of the article record. All identified records were independently screened by title and abstract by both authors. Unpublished manuscripts, conference abstracts, and other non-peer reviewed scientific correspondence were excluded from retrieval. The results of the independent searches were compared and collated in Excel (Microsoft, Redmond, WA, USA) to eliminate duplicate records. The full texts of records identified as potentially containing relevant data and variables were retrieved and assessed for inclusion.

### 2.2. Study Selection and Eligibility Criteria

If necessary, retrieved publications were translated using the translation tool in Adobe Acrobat (Adobe San Jose, CA, USA) or Google Translate (Google Mountain View, CA, USA) to facilitate eligibility review and data extraction. Retrieved studies were required to meet the following criteria to be eligible for inclusion: (i) the study utilized larval *Cx. pipiens* complex (*pipiens, quinquefasciatus,* or *pallens*), *Ae. Aegypti,* or *Ae. albopictus* laboratory colonies that were regarded by the authors to be a pesticide-susceptible control colony and maintained as such (no publications with F_1_ susceptible progeny), (ii) late-instar larval mosquitoes were exposed to methoprene or pyriproxyfen over a range of concentrations to develop mortality percentages in a bioassay experiment that included untreated controls, (iii) publications utilized a continuous exposure (exposure of larvae for ≥24 h) methodology that recorded mortality until all mosquitoes were dead or had emerged successfully, (iv) inhibition of emergence (IE) percentages for various exposures were corrected for control mortality and were analyzed using a probit-based methodology to calculate and report original IE_50_, IE_90_, and/or IE_95_ values. These criteria were constructed to include studies that are aligned with WHO IGR larval resistance testing protocols [17,18]. In addition, a publication was excluded if (i) it contained unoriginal reference IE values reprinted and/or cited from previous publications, or (ii) the control/reference mosquitoes were regarded as a pesticide-susceptible reference strain but were identified as F_1_ progeny or other field-collected mosquitoes.

### 2.3. Risk of Bias Evaluation

To assess the risk of bias, we evaluated study-level, outcome-level, and publication-level factors. Since our study aims to characterize the variability in published susceptible reference IE values derived from WHO-standardized IGR testing protocols, we designed our eligibility criteria to minimize study-level bias. We excluded publications with clear sources of variation, such as differences in bioassay design, exposure duration, assay length, and reference strain provenance. This approach ensured our study population closely aligned with WHO methodologies while also isolating experimental heterogeneity, biological variation, and other unknown confounders as the primary remaining sources of bias for characterization in this meta-analysis. Because all reported reference IE values were originally intended as experimental controls, the risk of reporting bias was considered negligible. This methodology enabled a synthesis of reference IE values under comparable experimental conditions, facilitated an analysis of heterogeneity within established protocols, and provided a clearer understanding of true susceptibility variation in mosquito populations to IGRs while minimizing known biases.

At the outcome-level, we evaluated the consistency of bioassay and probit regression methods for determining IE_50_, IE_90_, and IE_95_ values. To address redundancy bias from repetitive IE values in the literature, we excluded publications that reported unoriginal or previously published IE values. To reduce the risk of repeated measure bias, publications with multiple measurements on the same reference strain in the same publication were averaged to produce one set of reference values per publication. Publication bias was evaluated subjectively through funnel plots and objectively using Egger’s regression (Supplemental Figures S13–S15) [24].

Included publications were grouped by species and IGR into six distinct subgroups. Each IE value (IE_50_, IE_90_, and IE_95_) for each species/IGR pair was analyzed separately for a total of 18 analyses (3 species × 2 IGRs × 3 IE values). Because *Cx. pipiens* is a pan-global species complex composed of a variety of subspecies, forms, and hybrids, studies on *Culex* spp. were first grouped by subspecies to facilitate a subgroup analysis of *Cx. quinquefasciatus*, *pipiens*, and *pallens* to determine if treating the species complex as a single species was appropriate.

### 2.4. Data Extraction and Formatting

Both authors independently reviewed the retrieved records against the inclusion and exclusion criteria. M.C. extracted the data from the studies identified for inclusion. K.L. audited the data extraction process by comparing data records with the original publications. Any discrepancies were resolved through thorough discussion. No automation tools were used. Publications that met inclusion criteria were grouped by species/IGR pair for data extraction. The following data were extracted from eligible publications:

(1) Bibliographical information: author names, publication year, journal of publication, etc.
(2) Subject: mosquito species and subspecies, if applicable (for *Culex* spp.).
(3) Intervention: methoprene or pyriproxyfen exposure to larval mosquitoes in a dose/concentration-response bioassay including corrected mortality and probit regression.
(4) Outcomes: inhibition of emergence concentration point values (IE_50_, IE_90_, and/or IE_95_ values) as well as any uncertainty estimates, if available (95% CI, SD, SE, etc.).

All inhibition of emergence reference values were converted from their published units (ppb, µg/L, mg/L, etc.) to parts-per-million (ppm ±95% CI) and then Log_10_ transformed to stabilize variance prior to statistical analysis. We chose ppm to maintain consistency across studies and facilitate comparisons, as it is commonly used in larvicide efficacy research. While WHO guidelines often use µg/L, ppm is functionally equivalent (1 ppm = 1 mg/L = 1000 µg/L) and allows for uniformity in our meta-analysis without altering result interpretation. Confidence intervals extracted from the literature were also transformed into Log_10_ and used to estimate the SD for each IE value. When reported, SE was converted to SD. For publications that lacked reported uncertainty measurements (CI, SD, or SE), the SD was imputed by multiplying the Log_10_(SD) to Log_10_(IE) ratio for complete studies by the Log_10_(IE) value of incomplete studies (those studies requiring an approximated SD) following the method outlined in [25]. Imputed SDs were used for all further analyses for studies that did not include them.

### 2.5. Statistical and Sensitivity Analyses

The conversion of concentrations, Log_10_ transformations of data, and imputation of standard deviations (SDs) were performed using Microsoft Excel (Microsoft Inc., Redmond, WA, USA). Statistical analyses, including publication bias assessments (funnel plot and Egger’s regression), heterogeneity assessments, and sensitivity analyses, were conducted using the Meta-Essentials: Excel Workbooks for Meta-analysis tool [26].

The *Cx. pipiens* complex mosquitoes (*quinquefasciatus, pipiens,* and *pallens*) were analyzed in Meta-Essentials using an Analysis of Variance (ANOVA) test to determine if these subspecies groups were significantly different or if they could be treated as a single group for the purposes of computing an overall effect size. Heterogeneity was evaluated both within and across publications using Cochran’s Q, I^2^, T^2^, and T for all subgroups with more than 3 publications (16 out of 18 species/IGR/IE pairings). Descriptive statistics (minimum, maximum, interquartile range, median, and geometric mean with ± 95% confidence intervals) for each species/IGR/IE value pairing were generated using GraphPad Prism version 10 (GraphPad Software, Boston, MA, USA). Extracted data (IE_conc_ ± 95% CI) for each species/IGR/IE value pairing were made into a forest plot (Supplemental Figures S1–S6).

The overall effect size (composite IE values) for each subgroup/IGR pair/IE value was determined using a random-effects model in the Meta-Essentials Excel Workbook. This method calculates a combined effect size using inverse variance weighting and incorporates between-study variance through the DerSimonian–Laird (DL) estimator [26,27]. Ninety-five percentile confidence intervals for the DL combined effect values were calculated using a weighted variance methodology [28,29]. The DL combined effect value was calculated for each species (*Cx. pipiens* complex, *Ae. aegypti*, and *Ae. albopictus*), each reference IE value (IE_50_, IE_90_, and IE_95_), and each IGR (methoprene or pyriproxyfen), resulting in a total of 18 DL combined effect IE values with corresponding 95% confidence intervals.

To assess the robustness of the DL combined effect susceptible reference values, three sensitivity analyses were performed: (1) A ROUT outlier test [30] was applied to all species/IGR/IE pairings on log_10_ transformed values, using a Q value corresponding to a 1% false discovery rate. Identified outliers were removed, and the DL combined effect value was recalculated to assess their influence. (2) To evaluate potential systematic bias from studies requiring imputed confidence intervals, a second sensitivity analysis was conducted by excluding all values from such studies and recalculating the DL combined effect value. (3) The individual weights of each study used for the DL method were calculated and qualitatively assessed for homogeneity. (4) A geometric mean with 95% CIs was calculated for each species/IGR/IE value to compare against the DL inverse variance weighting methodology, providing an alternative measure of central tendency.

All DL combined effect values (*n* = 18) were assessed for certainty using a modified GRADE approach [31]. Risk of bias and indirectness were considered minimal for each subgroup since all included studies followed WHO protocols, and this meta-analysis focuses exclusively on control values from previous literature. DL combined effect values were downgraded if they exhibited high heterogeneity (I^2^ > 75%) and/or if their upper 95% confidence interval exceeded three times the DL combined effect value. If Egger’s regression indicated publication bias within a subgroup pairing, the certainty of the DL combined effect value was downgraded by one level accordingly. In the final analysis, the DL combined effect susceptible values were compared to the original published IE values to calculate a resistance ratio (published IE_50_ value/DL combined effect IE_50_ value). This analysis aims to identify the number and percentage of publications that may have inadvertently used potentially resistant mosquitoes as susceptible references.

## 3. Results

### 3.1. Literature Search and Study Selection Results

**Figure 1:**
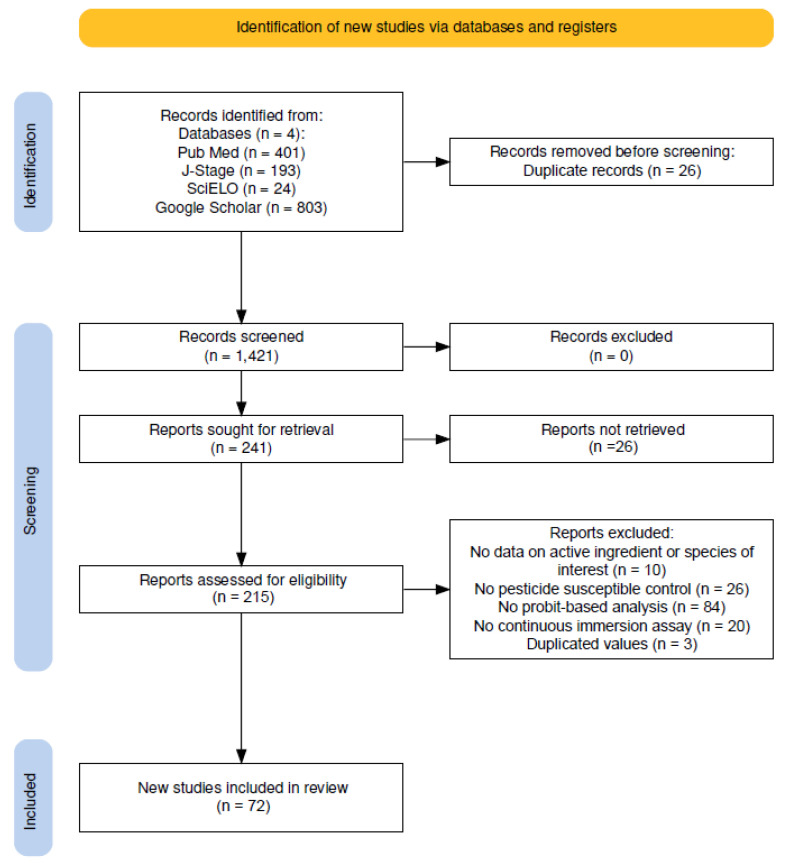
PRISMA flow chart of the publication assessment and inclusion process. No automation tools were used.

Our search strategy identified 1421 records across the four databases (401 from PubMed, 193 from J-STAGE, 24 from SciELO, and 803 from Google Scholar; Figure 1) for our six species/IGR pairings. After the titles and abstracts were reviewed for duplicate records, 241 reports were sought for retrieval. Of the 241 reports sought for retrieval, 26 studies contained data on multiple species or IGRs and were only retrieved once, yielding a total of 215 unique publications. An assessment for eligibility concluded that 84 were excluded for lacking a probit-based analysis of the mortality results, a further 10 were excluded for lacking IE value data on the species of interest, 26 were excluded for the use of a reference strain that did not meet inclusion criteria, 20 were excluded for not utilizing a continuous immersion design and, finally, 3 were excluded for reporting IE values from a previous publication. This yielded a total of 98 records from 72 unique publications that were eligible for inclusion (Figure 1). A total of 24 studies were identified that contained data for the *Cx. pipiens*/methoprene pairing [9,32,33,34,35,36,37,38,39,40,41,42,43,44,45,46,47,48,49,50,51,52,53,54], 17 for *Ae. aegypti*/methoprene [37,38,39,40,52,55,56,57,58,59,60,61,62,63,64,65], 8 for *Ae. albopictus*/methoprene [40,42,49,60,66,67,68,69], 12 for *Cx. pipiens*/pyriproxyfen [34,40,50,65,70,71,72,73,74,75,76,77], 26 for *Ae. aegypti*/pyriproxyfen [37,40,56,57,64,65,78,79,80,81,82,83,84,85,86,87,88,89,90,91,92,93,94,95,96] and, finally, 11 for *Ae. albopictus*/pyriproxyfen [40,66,68,69,89,90,97,98,99,100,101]. A forest plot of extracted IE values, as well as their associated confidence intervals (if reported) can be found in Supplemental Figures S1–S6 for all species/IGR/IE pairings.

### 3.2. Risk of Bias, Culex spp. Subgroup, and Heterogeneity Analysis Results

Since the inclusion criteria closely followed the WHO IGR larvicide test protocol and ensured that only studies meeting at least the minimum standards were included, and because the exclusion criteria were designed to isolate study-level and outcome-level bias from variability inherent in the protocol design, no additional exclusions of publications for bias were deemed necessary. An ANOVA subgroup analysis of *Cx. pipiens, quinquefasciatus,* and *pallens* indicated that there was no significant difference between these subgroups for either methoprene at IE_50_, IE_90_, or IE_95_, or pyriproxyfen at IE_50_ and IE_90_ (Table 1). As no significant differences in IE values were found for these species’ subgroups, the three *Culex* subspecies were treated as a single group for all subsequent analyses.

As demonstrated in Table 2, there was significant heterogeneity between studies. The I^2^ values for each published IE value ranged from 34.09% to 99.84% with 16 out of 18 subgroups exhibiting values above 75%, indicating that most of the heterogeneity resulted from differences between studies rather than from the variability found within studies. The pooled estimates (T) were found to range from 0.13 to 1.76, indicating that the true effect sizes across studies (10^T^) varied by a factor of between 1.34- and 57.97-fold (Table 2).

Funnel plots and an Egger’s regression were used to assess potential publication bias for each species/IGR/IE value pairing. As Table 3 demonstrates, only the *Ae. albopictus*/methoprene/IE_50_ exhibited a *p*-value < 0.05 (*p* = 0.043), indicating asymmetry in the funnel plot (Supplemental Figures S13–S15). The *Cx. pipiens*/pyriproxyfen/IE_90_ subgroup exhibited a *p*-value of 0.055, indicating a marginal asymmetry in the funnel plot (Figure S13E). The remaining subgroup pairings either did not have enough publications for us to conduct the regression (*n* = 2), or were not significant (*n* = 14).

### 3.3. Summary Statistics, Combined Effects Sizes, and Sensitivity Analysis

**Figure 2:**
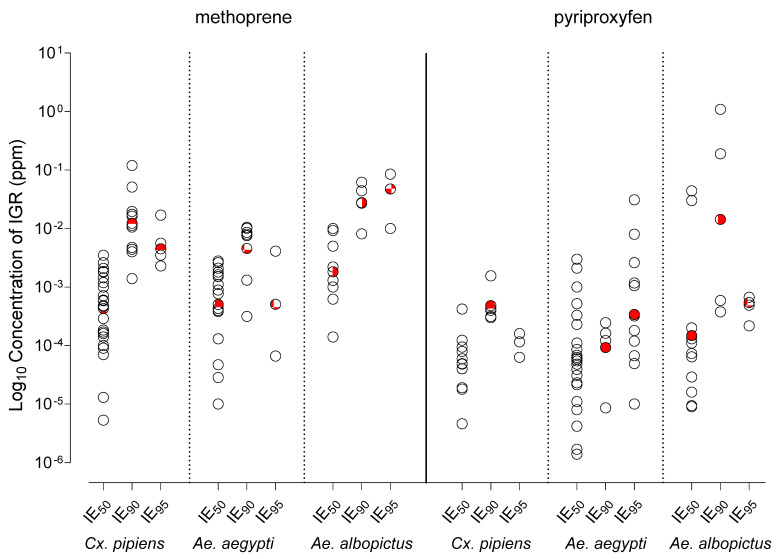
Distribution of IE_50_, IE_90_, and IE_95_ values (in ppm) reported in the literature (open circles), and DL combined effect values (red-filled circles).

The summary statistics for all species/IGR/IE combinations are presented in Table 4 (reported in ppb). As expected, the minimum, interquartile range (IQR), median, and maximum IE values increase from IE_50_ to IE_90_ across all species/IGR pairings. However, some species/IGR pairings demonstrate a decrease in concentration from IE_90_ to IE_95_. The DL combined effect value demonstrates that, for all species, pyriproxyfen is active at approximately a 10-fold lower concentration than methoprene at the IE_50_ value. In addition, the DL combined effect value for *Ae. albopictus* at IE_50_ demonstrates that this species is less sensitive to the IGRs examined in this study than either *Cx. pipiens* or *Ae. aegypti* (Table 4). Figure 2 illustrates the distribution of extracted IE values (open circle) as well as the DL combined effect value (red circle). In nearly all species/IGR/IE pairings, the DL combined effect value clustered towards the center of the distribution of published IE values. Also notable is that, in four out of six IE_95_ pairings, the DL combined effect value declined from the IE_90_ value.

Table 5 contains the results of three of the sensitivity analyses that were conducted. Most comparisons between the various analyses indicated little effect from studies that did not report confidence intervals or from studies that could be identified as outliers. In nearly all cases where a sensitivity analysis could be conducted, the combined effect size remained robust. However, in the *Ae. aegypti*/methoprene/IE_90_ pairing, the removal of two outliers doubled the DL combined effect value (from 4.559 ppb to 9.208 ppb), indicating that two studies were strongly influencing and lowering this IE_90_ value. In addition, the geometric means and 95% confidence interval for all species/IGR/IE pairings agreed well with those derived using the DL method. Supplemental Figures S7–S12 display the weighting of each publication within the DL method to create an overall combined effect size for each subgroup pairing. As these figures illustrate, there were no publications that exhibited an excessive weighting or influence on the DL combined effect value (Supplemental Figures S7–S12).

A certainty of evidence analysis was conducted for all species/IGR/IE value pairings (Table 6). The DL combined effect IE_50_ and IE_90_ values for methoprene were evaluated to be moderately certain for *Cx. pipiens* and *Ae. aegypti* and low to very low for *Ae. albopictus.* For pyriproxyfen, the IE_50_ value was again evaluated to be moderate for *Cx. pipiens* and *Ae. aegypti* and low for *Ae. albopictus.* The most common factor that caused a downgrade in the certainty was the high heterogeneity observed across studies, followed by imprecision (upper 95% confidence intervals more than 3× the DL combined effect value) (Table 6). One subgroup was downgraded for possible publication bias (*Ae. albopictus*/methoprene/IE_50_), and another exhibited marginal publication bias but was not downgraded (*p*-value = 0.055; *Cx. pipiens*/pyriproxyfen/IE_90_). In two cases, there were not enough publications to conduct an Egger’s regression nor enough publications to assess inconsistency (*Ae. aegypti*/methoprene/IE_95_ and *Ae. albopictus*/methoprene/IE_95_), and so these subgroups were similarly downgraded on both of these criteria (Table 7).

To assess how assumptions of susceptibility in the reviewed literature compare with the DL combined effect values from our meta-analysis, we calculated resistance ratios (RRs) using our DL combined effect value as a reference value. For *Ae. albopictus*, a comparison of our methoprene DL combined effect IE_50_ value against the original published susceptible reference values showed that 25% (two out of eight) of reference strains exhibited an RR > 5 (Table 7). For pyriproxyfen, 18% (2 out of 11) studies exhibited RRs > 10, indicating the presence of resistance in supposedly susceptible reference mosquitoes, while the remaining RRs were <5 (Table 7). For *Ae. aegypti*, the methoprene comparisons showed that 11.7% (2 out of 17) of reference strains had RRs > 5, with another 47% (8 out of 17) between 1 and 5 (Table 4). For pyriproxyfen, 12% (6 out of 26) studies exhibited RRs between 1 and 5, with another three exceeding RR 10 (Table 7). For *Cx. pipiens*, the methoprene pair showed only 8% (2 out of 24) studies with RRs >5, while the pyriproxyfen pair showed 8% (1 out of 12) with RRs > 5 (Table 7).

## 4. Discussion

### 4.1. Literature Characterization

In this review and meta-analysis, we systematically collected publications containing inhibition of emergence value data on reference vector mosquitoes that were considered susceptible by the study authors (summarized in Supplemental Figures S1–S6). To the best of our knowledge, this is the first comprehensive review and analysis of published reference values for the IGRs methoprene and pyriproxyfen. Reference IE values form the basis for nearly all dose/concentration-response mosquito resistance assessments, significantly influencing product selection, product rotation strategies, and broader mosquito management and public health decisions [18]. Therefore, characterizing and summarizing these reference values provides critical context for interpreting both historical and future reports of resistance in vector mosquitoes. From this meta-analysis, we identified several key findings: (1) Despite using eligibility criteria designed to exclude major sources of bias, the remaining literature still demonstrates a strikingly high degree of heterogeneity between studies. (2) Many experimental controls and design elements outlined in WHO and other standardized protocols may not be sufficient to ensure reliable resistance measurements across studies. (3) Some reference populations labeled as susceptible may not be truly susceptible. Consequently, resistance in field populations could go unnoticed, as inflated reference values may underestimate resistance ratios. (4) Finally, we provide DL combined effect IE_50_, IE_90_, and IE_95_ values for two IGRs and three mosquito species that can be used for future resistance evaluations, should a susceptible reference colony be inaccessible.

Heterogeneity in the published IE values, as characterized by I^2^ indices (Table 2), was consistently high—exceeding 75% in nearly all species/IGR/IE pairings where this metric could be calculated. This suggests that most variation in the dataset arises from differences between studies rather than within-study variability. The WHO protocol for larval resistance testing recommends conducting bioassays in triplicate, with untreated controls to correct mortality before fitting a probit regression. This level of replication and internal control within each study helps minimize within-study variability, improve probit regression accuracy, and narrow confidence intervals. However, while reducing within-study variability enhances precision, it can also amplify heterogeneity estimates that assess variability between studies; especially when unknown confounders or methodological discrepancies may exist.

Funnel plots and Egger’s regression are used to assess the relationship between effect size (in this case, logged IE value) and standard error. Completely unbiased literature based on the same sample population will produce results that vary randomly around the true effect size, creating a symmetrical funnel plot and a non-significant Egger’s regression. Selective reporting of results (publication bias) and/or the assessment of biologically different populations will produce a plot with systematic asymmetry. However, in this meta-analysis, we analyzed the controls (rather than the treatment effect) that were used as a baseline, mainly for observational studies. Because we are analyzing the baseline controls, the risk of publication bias and selective reporting with this type of data is implicitly lower. Only one subgroup pairing, *Ae. albopictus*/methoprene/IE_50_, demonstrated a significant *p*-value indictive of asymmetry (Table 3; *p* = 0.043). The *Cx. pipiens*/pyriproxyfen/IE_90_ pairing also exhibited a marginal asymmetry (Table 3; *p* = 0.055). These results suggest that the IE reference values analyzed in this meta-analysis are assessing distinct biological populations of *Ae. albopictus* rather than estimates of a single underlying effect and, thus, the asymmetry in the funnel plot may reflect true biological variability in reference colonies rather than publication bias. Additionally, the high heterogeneity observed in the dataset suggests that differences in mosquito strain susceptibility, rearing conditions, or testing methodologies is contributing to the observed asymmetry.

Although most studies measured their reference populations with high precision, variations in methodology, biological factors, and other unknown factors contributed to the high heterogeneity observed in IE values (Table 2). This heterogeneity persisted despite general adherence to WHO protocols, which are designed to enhance the accuracy and precision of IGR resistance testing. However, methodological differences—such as variations in larval rearing conditions, exposure durations, and test environments—may have influenced the observed variability. The certainty analysis of the DL combined effect values indicated that high heterogeneity led to a downgrade in the certainty of our values in 16 out of 18 subgroups (Table 7). Additionally, limited publication data reduced certainty in the *Ae. aegypti*/methoprene/IE_95_ and *Ae. albopictus*/methoprene/IE_95_ pairings. A lack of precision, defined as 95% confidence intervals exceeding three times the DL combined effect value, was observed in 10 out of 18 pairings. Overall, both *Cx. pipiens* and *Ae. aegypti* exhibited moderately certain DL combined effect values for IE_50_ and IE_90_. In contrast, the relative scarcity of studies on *Ae. albopictus*, coupled with high heterogeneity, resulted in low certainty estimates across most concentrations and IGRs. The outcome observed here highlights an urgent need for greater standardization in experimental design and more rigorous reporting of experimental conditions to improve cross-study comparisons.

### 4.2. Standardized Protocols and Methodological Variability

The WHO’s standardized IGR testing protocols, outlined in a series of publications [17,18], modify standard toxicity bioassays by replacing lethal dose (LD) or lethal concentration (LC) measurements with inhibition of emergence (IE_50_, IE_90_, and IE_95_) values, given the unique action of juvenoid IGRs. To properly apply a probit-based methodology to IGRs, several key adjustments and controls must be incorporated [17]. Since juvenoid IGRs disrupt metamorphosis rather than cause immediate toxicity, bioassays must monitor larvae until either successful emergence or death. During this period, larvae should be fed. WHO recommendations include finely ground yeast extract, rabbit pellets, or ground fish/mouse food [17]. Other standardized conditions include the use of untreated controls with mortality correction, use of either technical grade or formulated IGR stock solutions, environmental controls (a temperature of 25–28 °C and a 12 L:12 D photoperiod), use of disposable cups, glass bowls, or beakers for assays, and reference populations derived from either laboratory colonies or susceptible F_1_ progeny from field-collected mosquitoes [18]. Our inclusion criteria closely aligned with all WHO recommendations, allowing protocol variability, but deliberately excluded studies conducted with F_1_ progeny of field-collected mosquitoes.

Despite these standardized protocols, some of the observed interstudy variation likely stems from experimental details such as feeding regime, bioassay container material, temperature, and IGR source. For instance, pyriproxyfen is well adsorbed by organic materials, making it more likely to be ingested by larvae [97,102]. This suggests that the timing, method, and type of food used could influence both the bioavailability of the IGR and the amount ingested by larvae. In addition, bioassay container material significantly affects pyriproxyfen IE values, with variations of up to 150-fold across different materials (glass, concrete, rubber, polystyrene, etc.) [100]. Methoprene is similarly known to exhibit hydrophobic binding tendencies and would be expected to be vulnerable to the same biases [103]. Additionally, WHO protocols permit the use of both technical grade and formulated products, but formulated products can often include unreported solubility-enhancing agents or other adjuvants, potentially making their bioavailability difficult to compare with technical grade materials [104]. Both methoprene and pyriproxyfen are poorly soluble in water, and WHO recommendations suggest the use of either ethanol or acetone as a diluent (based on manufacturer recommendation) [17,105]. Both diluents are toxic to mosquitoes in higher concentrations, necessitating the dosing of small quantities of diluted active ingredients, further complicating bioassay standardization [17].

Although WHO protocols attempt to control temperature and photoperiod, both of which can influence the effects and degradation of IGRs [48,106,107], minor variations in these parameters may still be introducing significant sources of heterogeneity. We did not attempt to subgroup and analyze studies based on these factors because some experimental conditions were inconsistently reported in the literature, precluding further subgroup analysis, and much of the variability in experimental conditions is explicitly allowed under WHO protocols. Each of the aforementioned factors may be exhibiting an effect on the determination of susceptible IE values, which may not matter much within a well-controlled study but may hinder interstudy comparisons or the identification of regional trends or patterns.

### 4.3. Biological Variability in Reference Strains

While minor methodological differences may exist across studies, the most likely source of between-study variability is the inherent biological differences in reference mosquito strains. Even after excluding studies using the progeny of recently field-collected mosquitoes (F_1_) with an unknown susceptibility status, high variability persisted (Table 2). A qualitative review of eligible studies reveals that reference strains originated from commercial suppliers, universities, mosquito control districts, government laboratories, and other entities. Some susceptible strains have been laboratory-maintained for decades (e.g., Rockefeller strain, *Ae. aegypti*), while others were recently colonized following global invasions and likely experienced exposure to pesticides or other chemistries prior to captivity (e.g., ATM95, *Ae. albopictus*). In some cases, strains were generically labeled as “pesticide-susceptible” or assigned conflicting or unclear names (e.g., SLAB vs. University of Montpellier), making it difficult to determine their provenance. In addition, the literature used in this meta-analysis was derived primarily from the United States, Japan, and, to a lesser degree, Southeast Asia, the Caribbean, and South America. It is unknown what effect this geographic clustering of the primary literature may be exhibiting on the various parameters assessed in this meta-analysis.

Since *Culex* subspecies were clearly described in all included publications, we evaluated whether subspecies-level variation could explain the observed heterogeneity, at least for *Cx. pipiens* complex IE values. We sub-grouped the *Cx. pipiens* species complex (*pipiens, quinquefasciatus*, and *pallens*), and there were no significant differences between subspecies groups (Table 1). Moreover, in all comparisons, the F-statistic was <1, indicating that within-subspecies-group variance exceeded between-subspecies-group variance (Table 1). This result suggests that the source of reference *Culex* spp. mosquitoes was more important than their taxonomic designation and highlights how biological variation within reference colonies may be an underappreciated confounding factor in resistance assessments. A similar analysis of the potential of biological variability to be the cause of high heterogeneity for *Ae. aegypti* and *Ae. albopictus* was not conducted due to a lack of consistent information that could enable a subgroup comparison. However, the high heterogeneity observed for these species (Table 2) indicates that variability—either biological or methodological—exists.

A comparison of *Cx. pipiens* and *Ae. aegypti* DL combined effect IE_50_ values for methoprene and pyriproxyfen shows remarkable similarity (0.428 ppb vs. 0.505 ppb for methoprene and 0.048 ppb vs. 0.053 ppb for pyriproxyfen; Table 3 and Table 4). However, *Ae. albopictus* exhibited DL combined effect IE_50_ values that were approximately threefold higher than those of *Cx. pipiens* and *Ae. aegypti* (Table 3). Notably, over 60% of studies on *Ae. albopictus*/methoprene and over 90% on *Ae. albopictus*/pyriproxyfen were published after the year 2000, by which time this species had already invaded most continents [108,109]. This suggests that the relatively recent global invasion of *Ae. albopictus* may have resulted in laboratory colonies being established from source populations that were less susceptible than originally assumed. Alternatively, it is possible that *Ae. albopictus* exhibits some undescribed constitutive features that result in lower susceptibility to IGRs. In addition, there were comparatively fewer studies that examined *Ae. albopictus*, which may contribute to these differences and reduce the robustness of our DL combined effect values for this species. Future resistance assays with *Ae. albopictus* should pay closer attention to the generated susceptible reference values, as seemingly small increases in susceptible reference value can have disproportionate effects on the resultant RR, thus obscuring detections of resistance.

As Table 5 shows, there are a variety of publications where the susceptible reference strains should be regarded as “moderately resistant” or “highly resistant” under WHO criteria (RR 5–10 or RR > 10) when compared to the susceptible DL combined effect value we generated in the meta-analysis [18]. In the most extreme examples, the *Ae. albopictus*/pyriproxyfen pair resulted in two publications with an RR > 200. In the *Ae. aegypti*/pyriproxyfen pair, two studies exceeded an RR of 20. It is important to note that, since susceptible reference values form the denominator in the resistance ratio calculation, any increase in this denominator results in a non-linear inverse (hyperbolic) decrease in RR, potentially obscuring resistance detection. Therefore, published reference values that exceed our DL combined effect value by a ratio of between 1 and 5, which seems moderate, are still considered “susceptible” under WHO definitions and may disproportionately and dramatically skew RR calculations lower, potentially underestimating resistance.

Together, the results presented in Table 5 strongly suggest that laboratory strains of *Ae. aegypti* and *Ae. albopictus* (and, to a lesser degree, *Cx. pipiens*) should not be assumed to be susceptible, despite a presumed lack of pesticide exposure or an unbroken laboratory lineage. Given the stenogamous nature of *Aedes* spp. mosquitoes, inadvertent admixture between lab and field strains in insectaries may have also contributed to the variability observed in these colonies. Alternatively, pesticide exposure prior to colony establishment, cross-resistance between pesticide or chemical classes, or unintended pesticide exposures within laboratories may be more common than previously recognized. Finally, it is possible that founder effects or genetic bottlenecking have isolated reference strains from each other, making cross-study comparisons much more difficult. Future resistance assessments should strongly consider alternative methods to verify the susceptibility of their reference strains—either by comparing them to the values generated here or through independent validation methods.

### 4.4. Combined Effect Values

One goal of this meta-analysis is to integrate data from multiple bioassays to establish a more robust benchmark for future resistance evaluations. Table 3 presents summary statistics and the DL combined effect IE values with 95% confidence intervals. Due to high between-study variability and low within-study variability, the weights assigned to individual studies in the DL model were overall very similar between studies (Supplemental Figures S4–S6). There were no studies that exerted a disproportionate weight on the DL combined effect value. The sensitivity analyses conducted indicate that neither studies requiring imputation of confidence intervals, nor those with outliers removed, exerted much of an effect on the DL combined effect values, except for the *Ae. aegypti*/methoprene/IE_90_ pairing, where two values identified as outliers were exerting a large effect on the resulting DL combined effect value (Table 4). When taken together, these results indicate that, for most species/IGR/IE pairings, we were able to generate moderately robust susceptible reference values for use as a comparison or to validate other reference values.

With all studies analyzed together, the DL combined effect values for IE_50_, IE_90_, and IE_95_ tended to fall around the center of the distribution of values extracted from the literature and were remarkably similar to the geometric mean (Figure 2). A comparison between the geometric mean and DL combined effect value also demonstrates that within-study variation had minimal influence on the DL combined effect values (Table 4). Given that both our DL combined effect value and geometric mean values are well-centered within a wide range of original values, and that small increases in the IE reference values can disproportionately decrease the resulting RR when assessing resistance, the DL combined effect and/or geometric mean values likely represent the upper bound of what should be considered truly susceptible reference mosquitoes. In addition, the lack of sensitivity of the DL combined effect values to outliers and other potential biases suggests that the values generated here, especially at IE_50_, are reasonable estimates that can be used to compare against a wide range of experimental conditions.

The summaries presented in Table 3 represent a composite of all published IE values and can serve multiple purposes: (1) assessing the continued susceptibility of existing reference strains, (2) assessing the susceptibility of F_1_ field-collected strains intended for use as references, and (3) providing a potential benchmark to accompany WHO larval IGR resistance protocols. However, since the source values used to derive composite values (geometric mean or DL combined effect) vary by orders of magnitude, these statistics should always be interpreted alongside their 95% confidence intervals when calculating resistance ratios. In other words, any resistance ratios derived from composite values in Table 3 or Table 4 should be reported as a range that includes both the lower and upper confidence limits rather than as a point estimate.

Though we report composite DL combined effect IE_90_ and IE_95_ values, the number of studies that reported these values was extremely limited, resulting in less robust estimates that suffered from inconsistency and imprecision and were downgraded accordingly (Table 6). In addition, IE_90_ and IE_95_ values are derived from the extreme end of the probit curve, making them more susceptible to heterogeneity in the sampled population compared to IE_50_ values. As a result, these higher IE values exhibit a wider distribution in the literature, which in turn lowers the certainty of the DL combined effect as assessed in Table 6. Table 3 indicates that IE_95_ values frequently decreased from IE_90_ values, contrary to the usual expectation of an increase, in part due to a lack of included values. Indeed, caution should be exercised in interpreting IE_95_ values for all pairings except for Ae. aegypti/pyriproxyfen. Various other species/IGR/IE pairings also exhibit “low” or “very low” certainty (Table 6). This suggests that, while a DL combined effect was computable, the addition of new evidence would likely have a noticeable impact on this value. In contrast, IE values assessed as “moderately” certain are less likely to be influenced by new evidence. In any case, all DL combined effect values have been reported with confidence intervals (with low-certainty estimates necessarily having wider confidence intervals) and should be regarded as a range rather than a point estimate to account for differences in the certainty of the estimate.

In place of comparing to the DL combined effect IE_90_ and IE_95_ values, summary statistics such as medians and the IQR could also be used for assessment. Inclusion of IE_90_ values, as recommended by the WHO, is necessary in future susceptibility evaluations. A large gap between IE_50_ and IE_90_ values can be indicative of resistance in a small portion of the population. In the case of maintaining susceptible mosquito colonies, this is a critical piece of evidence for potential accidental exposure to insecticides or underlying residual resistance after field colonization.

### 4.5. Statistical Considerations

Probit regression remains the standard toxicological method for quantifying susceptibility and resistance to pesticides, as it captures the non-linear dose/concentration-response relationship inherent in bioassays [110,111]. However, there are statistical considerations and interpretations that are often ignored. Although our inclusion criteria for this meta-analysis included the use of a probit analysis methodology for calculating IE values, we did not evaluate model fit nor require the inclusion of confidence intervals. Without including a statistical test to demonstrate the fit of the data, it is possible that the bioassay data used do not fit a probit distribution, leading to incorrect IE values and wider confidence intervals. The most common ways to evaluate regression fit are Pearson’s χ^2^ goodness-of-fit test, an alternative χ^2^ test, or evaluating the H value (χ^2^/df). The potential for poorly fitted probit regressions may be an additional level of variability within this meta-analysis. Furthermore, comparing confidence intervals can be used in place of or in addition to calculating a resistance ratio for interpreting susceptibility differences between populations. A ratio test can be used to determine if the results from two probit analyses are significantly different, rather than just reporting the fold change in IE values [112]. These additional statistical elements, recommended by the WHO [18], need to be reported in future studies.

We acknowledge that some of the studies included were conducted before the availability of modern computing equipment or performed on log-probit paper; thus, calculating confidence intervals and model fit was too cumbersome or inaccessible. However, with modern statistical software, probit analyses complete with confidence intervals and fit assessment should be the standard. R studio, Microsoft Excel, SPSS, Polo, Python, and SAS are commonly used statistical programs or languages that provide these outputs, some of which are completely free to download and use.

### 4.6. Recommendations and Future Directions

Resistance monitoring in vector mosquitoes is a critical component of effective mosquito management, and WHO protocols provide an indispensable framework for conducting standardized resistance assessments. However, uncharacterized confounders—such as experimental differences and inherent biological variations in colony populations—can obscure resistance patterns and complicate cross-study comparisons. Without reliable assessments or clear study-to-study comparisons, identifying trends in resistance becomes challenging, ultimately impacting the ability of public health and vector control agencies to manage mosquito populations effectively.

The goal of this review and meta-analysis was to better understand the variability in reported IE values and to establish benchmark values that help researchers assess the reliability of their findings. Additionally, this study aimed to highlight potential shortcomings in current experimental methods that may contribute to inconsistent results. Based on our findings, several recommendations can be made:

1. Existing protocols should be more detailed. For example, the use of disposable bioassay containers (such as polystyrene cups) should be universally recommended, along with a standardized larval feeding regime. Any experimental conditions left open to interpretation should be reevaluated and defined as precisely as possible.
2. Reference colonies should be periodically assessed for continued susceptibility, either through comparisons with other reference colonies or against the benchmark values reported here. No assumptions about susceptibility or pesticide exposure should be made.
3. The source of reference mosquitoes and any ongoing susceptibility assessments should be more thoroughly documented in the literature.
4. If F_1_ progeny of field-collected mosquitoes (or other field-collected mosquitoes) will be used as susceptible references, then they should be compared against the benchmarks generated here to assess the applicability of these reference values prior to any resistance determinations.
5. All bioassay probit analyses should report IE_50_, IE_90_, a goodness-of-fit evaluation, and confidence intervals for each mosquito population evaluated.

## Figures and Tables

**Table 1:** Summary of ANOVA results for the subgroup analysis of *Culex* spp. complex mosquitoes. A *p*-value for pyriproxyfen at IE_95_ could not be computed due to the low number of available studies.

methoprene			
*Culex pipiens*			
	IE_50_	IE_90_	IE_95_
Between groups (SS)	2.34	0.00	0.13
Within groups (SS)	25.14	13.12	2.46
Number of studies	24	12	4
F-statistic	0.09	0.00003	0.05108
Between groups (*df*)	2	1	1
Within groups (*df*)	21	10	2
Between groups *p*-value	0.31	0.99	0.72
pyriproxyfen			
	IE_50_	IE_90_	IE_95_
Between groups (SS)	4.57	0.77	0.00
Within groups (SS)	13.15	2.38	1.68
Number of studies	12	5	3
F-statistic	0.35	0.32	0.00
Between groups (*df*)	2	1	0
Within groups (*df*)	9	3	2
Between groups *p*-value	0.10	0.38	—

**Table 2:** Summary of heterogeneity across studies (Cochrane’s Q, I^2^, T^2^, and T) for each IGR/species/EI value pairing with >2 publications. T represents the standard deviation of true effect sizes on the log scale, and unlogged T represents the fold change in effect sizes across studies (10^T^).

methoprene									
	*Culex pipiens*			*Aedes aegypti*			*Aedes albopictus*		
	IE_50_	IE_90_	IE_95_	IE_50_	IE_90_	IE_95_	IE_50_	IE_90_	IE_95_
Q	736.52	103.15	4.24	6807.87	245.27	—	1183.47	201.80	—
p_q_	0.00	0.00	0.24	0.00	0.00	—	0.00	0.00	—
*df*	23	11	3	16	7	—	7	3	—
I^2^	96.88%	89.34%	29.32%	99.76%	97.15%	—	99.41%	98.51%	—
T^2^	0.34	0.17	0.01	1.13	0.20	—	0.38	0.20	—
T	0.58	0.41	0.08	1.06	0.44	—	0.62	0.44	—
Fold change in effect size (10^T^)	3.83	2.57	1.20	11.59	2.78	—	4.17	2.79	—
pyriproxyfen									
	IE_50_	IE_90_	IE_95_	IE_50_	IE_90_	IE_95_	IE_50_	IE_90_	IE_95_
Q	2373.51	120.11	18.96	11,381.13	567.01	5113.51	6448.14	521.46	3.03
p_q_	0.00	0.00	0.00	0.00	0.00	0.00	0.00	0.00	0.22
*df*	11	4	2	25	4	12	10	3	2
I^2^	99.54%	96.67%	89.45%	99.78%	99.29%	99.77%	99.84%	99.42%	34.09%
T^2^	0.13	0.15	0.06	0.19	0.52	0.45	1.57	3.11	0.02
T	0.36	0.39	0.24	0.44	0.72	0.67	1.25	1.76	0.13
Fold change in effect size (10^T^)	2.29	2.45	1.75	2.73	5.27	4.72	17.93	57.97	1.34

**Table 3:** Results of Egger’s regression on all species/IGR/IE pairings. Only the *Ae. albopictus*/methoprene/IE_50_ pairing was significant (*p* = 0.043). The *Cx. pipiens*/pyriproxyfen/IE_90_ pairing had a *p*-value of 0.055, indicating marginal funnel plot asymmetry. All other funnel plots were symmetrical.

methoprene									
	*Culex pipiens*			*Aedes aegypti*			*Aedes albopictus*		
	IE_50_	IE_90_	IE_95_	IE_50_	IE_90_	IE_95_	IE_50_	IE_90_	IE_95_
Intercept	0.99	0.23	1.21	9.86	−5.98	11.69	−16.24	2.89	−1.59
SE	2.19	2.26	1.09	8.79	5.11	−	6.35	14.15	−
*p*-value	0.658	0.922	0.382	0.279	0.286	−	0.043	0.857	−
Lower 95% CI	−3.55	−4.75	−2.26	−8.76	−18.07	−	−31.26	−42.14	−
Upper 95% CI	5.53	5.21	4.68	28.48	6.11	−	−1.22	47.92	−
*t*-test	0.45	0.10	1.11	1.12	−1.17	−	−2.56	0.20	−
pyriproxyfen									
	IE_50_	IE_90_	IE_95_	IE_50_	IE_90_	IE_95_	IE_50_	IE_90_	IE_95_
Intercept	−0.22	−12.82	10.91	1.98	34.20	9.51	9.73	−7.93	−1.55
SE	6.15	4.18	7.35	5.03	18.08	8.90	14.24	15.87	0.63
*p*-value	0.972	0.055	0.377	0.698	0.155	0.308	0.511	0.667	0.244
Lower 95% CI	−13.76	−24.43	−20.72	−8.38	−15.99	−9.89	−21.99	−58.44	−4.25
Upper 95% CI	13.32	−1.20	42.54	12.33	84.39	28.91	41.45	42.59	1.14
*t*-test	−0.04	−3.06	1.48	0.39	1.89	1.07	0.68	−0.50	−2.48

**Table 4:** Summary statistics (minimum, maximum, and IQR), as well as DerSimonian and Laird (DL) combined effects values ±95% CI for the IGRs pyriproxyfen and methoprene and the species *Cx. pipiens*, *Ae. aegypti*, and *Ae. albopictus* at IE_50_, IE_90_, and/or IE_95_.

methoprene										
		*Culex pipiens*			*Aedes aegypti*			*Aedes albopictus*		
		IE_50_ (ppb)	IE_90_ (ppb)	IE_95_ (ppb)	IE_50_ (ppb)	IE_90_ (ppb)	IE_95_ (ppb)	IE_50_ (ppb)	IE_90_ (ppb)	IE_95_ (ppb)
Number of studies		24	12	4	17	8	2	8	4	2
Minimum		0.005	1.390	2.290	0.010	0.314	0.066	0.140	8.100	10.000
	25% Percentile	0.145	4.490	2.593	0.255	2.867	0.066	0.715	12.830	10.000
	Median	0.532	13.760	4.550	0.889	7.789	2.083	1.750	35.500	47.500
	75% Percentile	1.378	19.020	14.080	1.712	9.697	4.100	8.150	57.500	85.000
Maximum		3.500	119.000	16.900	2.780	10.500	4.100	10.000	62.000	85.000
DL Combined Effect		0.429	12.316	4.502	0.505	4.559	0.507	1.818	27.689	47.285
	Lower CI (95%)	0.232	5.852	2.617	0.213	1.595	2.06 × 10^−12^	0.537	6.684	2.56 × 10^−4^
	Upper CI (95%)	0.790	25.920	7.746	1.201	13.033	1.25 × 10^11^	6.154	114.705	8.75 × 10^6^
pyriproxyfen										
		*Culex pipiens*			*Aedes aegypti*			*Aedes albopictus*		
		IE_50_ (ppb)	IE_90_ (ppb)	IE_95_ (ppb)	IE_50_ (ppb)	IE_90_ (ppb)	IE_95_ (ppb)	IE_50_ (ppb)	IE_90_ (ppb)	IE_95_ (ppb)
Number of studies		12	5	3	26	5	13	11	4	3
Minimum		0.005	0.300	0.063	0.001	0.009	0.010	0.009	0.376	0.217
	25% Percentile	0.018	0.306	0.063	0.019	0.065	0.058	0.016	0.429	0.217
	Median	0.045	0.400	0.160	0.054	0.162	0.320	0.073	94.790	0.490
	75% Percentile	0.090	0.990	0.160	0.254	0.203	2.150	0.200	861.800	0.668
Maximum		0.420	1.550	0.160	3.000	0.245	31.000	44.000	1086.000	0.668
DL Combined Effect		0.048	0.482	0.116	0.055	0.092	0.339	0.148	14.330	0.543
	Lower CI (95%)	0.025	0.206	0.030	0.023	0.017	0.080	0.021	0.024	0.176
	Upper CI (95%)	0.095	1.126	0.447	0.128	0.494	1.437	1.053	8713.355	1.671

**Table 5:** Sensitivity analyses for each species/IGR/IE pairing. Sensitivity was assessed in three ways: (1) By removing studies with imputed 95%CIs and recalculating the combined effect size, (2) by identifying and removing outliers and recalculating the combined effect size, and (3) by calculating a geometric mean with 95% CIs. If no studies were imputed or no outliers were detected, a new combined effect size was not calculated.

methoprene											
			*Culex pipiens*			*Aedes aegypti*			*Aedes albopictus*		
			IE_50_ (ppb)	IE_90_ (ppb)	IE_95_ (ppb)	IE_50_ (ppb)	IE_90_ (ppb)	IE_95_ (ppb)	IE_50_ (ppb)	IE_90_ (ppb)	IE_95_ (ppb)
Total number of studies			24	12	4	17	8	2	8	4	2
	DL combined effect		0.429	12.316	4.502	0.505	4.559	0.507	1.818	27.689	47.285
		Lower CI (95%)	0.232	5.852	2.617	0.213	1.595	2.06 × 10^−12^	0.537	6.684	2.56 × 10^−4^
		Upper CI (95%)	0.790	25.920	7.746	1.201	13.033	1.25 × 10^11^	6.154	114.705	8.75 × 10^6^
Imputed studies removed											
	Number of studies removed		12	4	0	6	1	0	2	0	0
	DL combined effect		0.485	10.479	−	0.348	4.227	−	2.404	−	−
		Lower CI (95%)	0.183	3.619	−	0.108	1.223	−	0.445	−	−
		Upper CI (95%)	1.282	30.341	−	1.117	14.612	−	12.980	−	−
Outliers removed											
	Number of studies removed		0	0	0	1	2	0	0	0	0
	DL combined effect		−	−	−	0.660	9.208	−	−	−	−
		Lower CI (95%)	−	−	−	0.318	8.005	−	−	−	−
		Upper CI (95%)	−	−	−	1.372	10.592	−	−	−	−
Geometric mean			0.365	11.910	5.248	0.504	4.507	0.520	1.808	27.790	29.150
		Lower CI (95%)	0.184	5.577	1.334	0.212	1.560	2.11 × 10^−15^	0.536	6.745	3.63 × 10^−5^
		Upper CI (95%)	0.726	25.450	20.650	1.197	13.020	1.28 × 10^8^	6.096	114.500	2.34 × 10^7^
pyriproxyfen											
			*Culex pipiens*			*Aedes aegypti*			*Aedes albopictus*		
			IE_50_ (ppb)	IE_90_ (ppb)	IE_95_ (ppb)	IE_50_ (ppb)	IE_90_ (ppb)	IE_95_ (ppb)	IE_50_ (ppb)	IE_90_ (ppb)	IE_95_ (ppb)
Total number of studies			12	5	3	26	5	13	11	4	3
	DL combined effect		0.048	0.482	0.116	0.055	0.092	0.339	0.148	14.330	0.543
		Lower CI (95%)	0.025	0.206	0.030	0.023	0.017	0.080	0.021	0.024	0.176
		Upper CI (95%)	0.095	1.126	0.447	0.128	0.494	1.437	1.053	8713.355	1.67
Imputed studies removed											
Number of studies removed			3	1	1	5	1	1	0	0	0
	DL combined effect		0.050	0.542	0.098	0.050	0.072	0.294	−	−	−
		Lower CI (95%)	0.020	0.170	0.000	0.019	0.007	0.063	−	−	−
		Upper CI (95%)	0.126	1.725	36.478	0.133	0.701	1.385	−	−	−
Outliers removed											
	Number of studies removed		0	1	0	0	0	0	0	0	0
	DL combined effect		−	0.347	−	−	−	−	−	−	−
		Lower CI (95%)	−	0.266	−	−	−	−	−	−	−
		Upper CI (95%)	−	0.453	−	−	−	−	−	−	−
Geometric mean			0.044	0.478	0.117	0.053	0.092	0.331	0.147	14.590	0.414
		Lower CI (95%)	0.021	0.207	0.031	0.023	0.017	0.08	0.021	0.024	0.098
		Upper CI (95%)	0.091	1.106	0.446	0.123	0.494	1.43	1.043	8964.000	1.753

**Table 6:** Certainty of evidence assessment for each species/IGR/IE value pairing. Subgroup pairings with I^2^ greater than 75% were downgraded. Subgroup pairings with a 95% UCI more than 3-fold the DL combined effect values were downgraded (downgrade denoted by “↓”). ↓* = no I^2^ value could be calculated due to the small number of publications included. ↓^†^ = no Egger’s regression could be conducted due to the small number of publications included. ↓^‡^ = *p*-value of 0.055 indicates the possibility of publication bias; subgroup was not downgraded.

methoprene									
	*Culex pipiens*			*Aedes aegypti*			*Aedes albopictus*		
	IE_50_	IE_90_	IE_95_	IE_50_	IE_90_	IE_95_	IE_50_	IE_90_	IE_95_
Risk of bias	—	—	—	—	—	—	—	—	—
Inconsistency (I^2^ > 75%)	↓	↓	—	↓	↓	↓*	↓	↓	↓*
Imprecision (UCI > 3 × IE)	—	—	—	—	—	↓	↓	↓	↓
Indirectness	—	—	—	—	—	—	—	—	—
Publication Bias (*p* < 0.05)	—	—	—	—	—	↓^†^	↓	—	↓^†^
Final Certainty Rating	Moderate	Moderate	High	Moderate	Moderate	Very Low	Very Low	Low	Very Low
pyriproxyfen									
	IE_50_	IE_90_	IE_95_	IE_50_	IE_90_	IE_95_	IE_50_	IE_90_	IE_95_
Risk of bias	—	—	—	—	—	—	—	—	—
Inconsistency (I^2^ > 75%)	↓	↓	↓	↓	↓	↓	↓	↓	—
Imprecision (UCI > 3 × IE)	—	—	↓	—	↓	↓	↓	↓	↓
Indirectness	—	—	—	—	—	—	—	—	—
Publication Bias (*p* < 0.05)	—	↓^‡^	—	—	—	—	—	—	—
Final Certainty Rating	Moderate	Moderate	Low	Moderate	Low	Low	Low	Low	Moderate

**Table 7:** Summary of RR IE_50_ for included publications. Resistance ratios were calculated by dividing the reference IE_50_ values reviewed here with the DL combined effect IE_50_ value.

Number (%) of Publications at RR IE_50_												
	*Culex pipiens*				*Aedes aegypti*				*Aedes albopictus*			
RR	<1	1–5	5–10	>10	<1	1–5	5–10	>10	<1	1–5	5–10	>10
methoprene	10 (42%)	12 (50%)	2 (8%)	0 (0%)	7 (41%)	8 (47%)	2 (12%)	0 (0%)	4 (50%)	2 (25%)	2 (25)%	0 (0%)
pyriproxyfen	7 (58%)	4 (33%)	1 (8%)	0 (0%)	13 (50%)	7 (27%)	3 (12%)	3 (12%)	8 (73%)	1 (9%)	0 (0%)	2 (18%)

## Data Availability

Data extracted from included studies is available upon reasonable request. Data used for all analyses is available upon reasonable request.

## Supplementary Materials

The following supporting information can be downloaded at: https://www.mdpi.com/article/10.3390/tropicalmed10040087/s1, Figures S1–S6: Forest plots of species/IGR values reported in the literature. Figures S7–S12: Weighting of each study at IE_50_, IE_90_, and IE_95_. Figures S13–S15: Funnel plots of published IE values.

## Abbreviations

The following abbreviations are used in this manuscript:

ANOVA	Analysis of Variance
CI	Confidence interval
DL	DerSimonian and Laird
IE	Inhibition of emergence
IGR	Insect growth regulator
IQR	Interquartile range
SD	Standard deviation
SE	Standard error
RR	Resistance ratio
WHO	World Health Organization.
