# Supplementary material for: Assessing Insect Growth Regulator Resistance Using Bioassays: A Systematic Review and Meta-Analysis of Methoprene and Pyriproxyfen Inhibition of Emergence in Three Vector Mosquito Species
Source: Trop Med Infect Dis. 2025 Mar 28;10(4):87. doi: 10.3390/tropicalmed10040087 (PMC12031331; doi:10.3390/tropicalmed10040087)
Supplement: Supplementary file 1 [file tropicalmed-10-00087-s001.zip › tropicalmed-3527225 revision supplementary.pdf]

Supplemental Figures

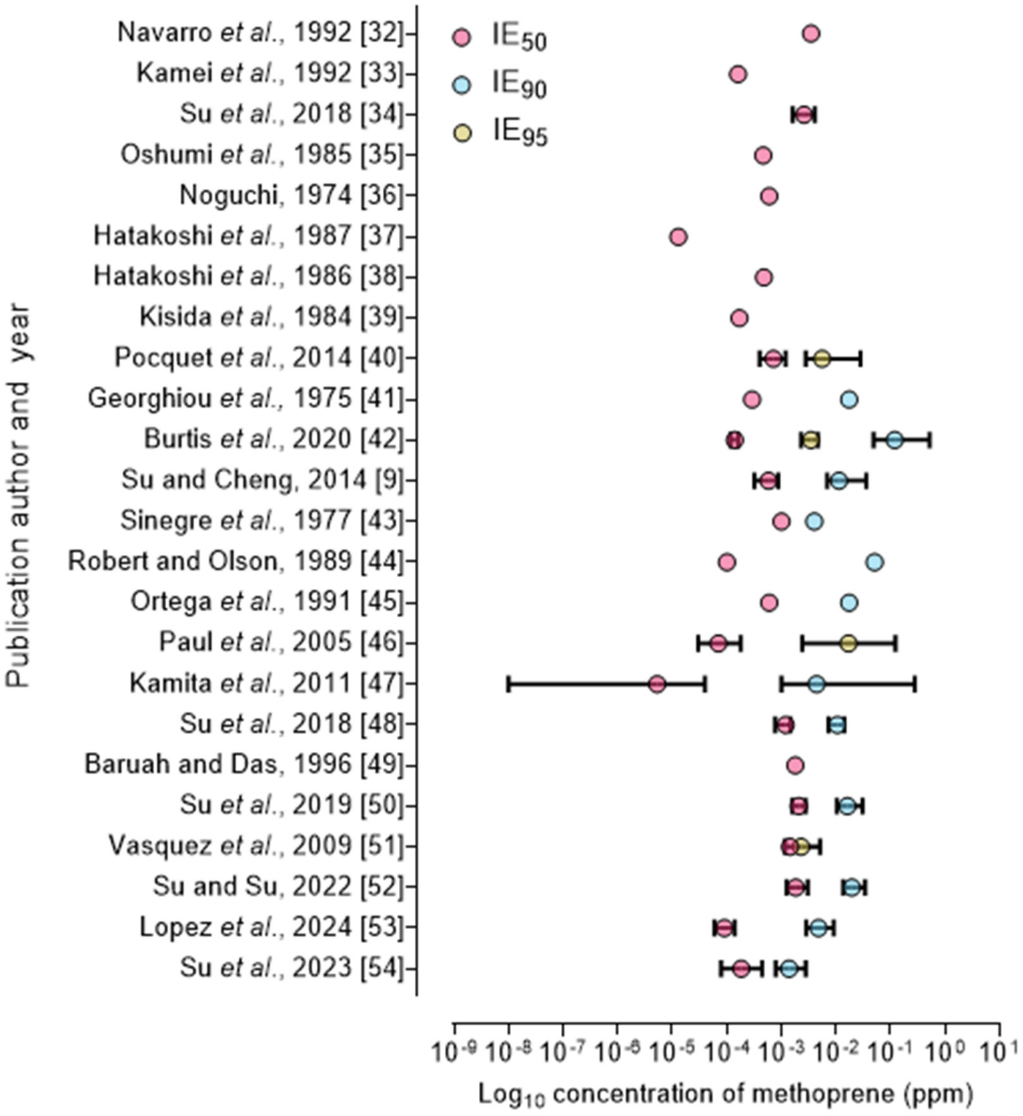

Figure S1. Forest plot of the *Cx. pipiens*/methoprene values reported in the literature converted to ppm (Log10). IE_{50} values are reported in red (n=24), IE_{90} in blue (n=12) and IE_{95} in yellow (n=4). Confidence intervals (95%) are displayed for publications where uncertainty measurements were reported.

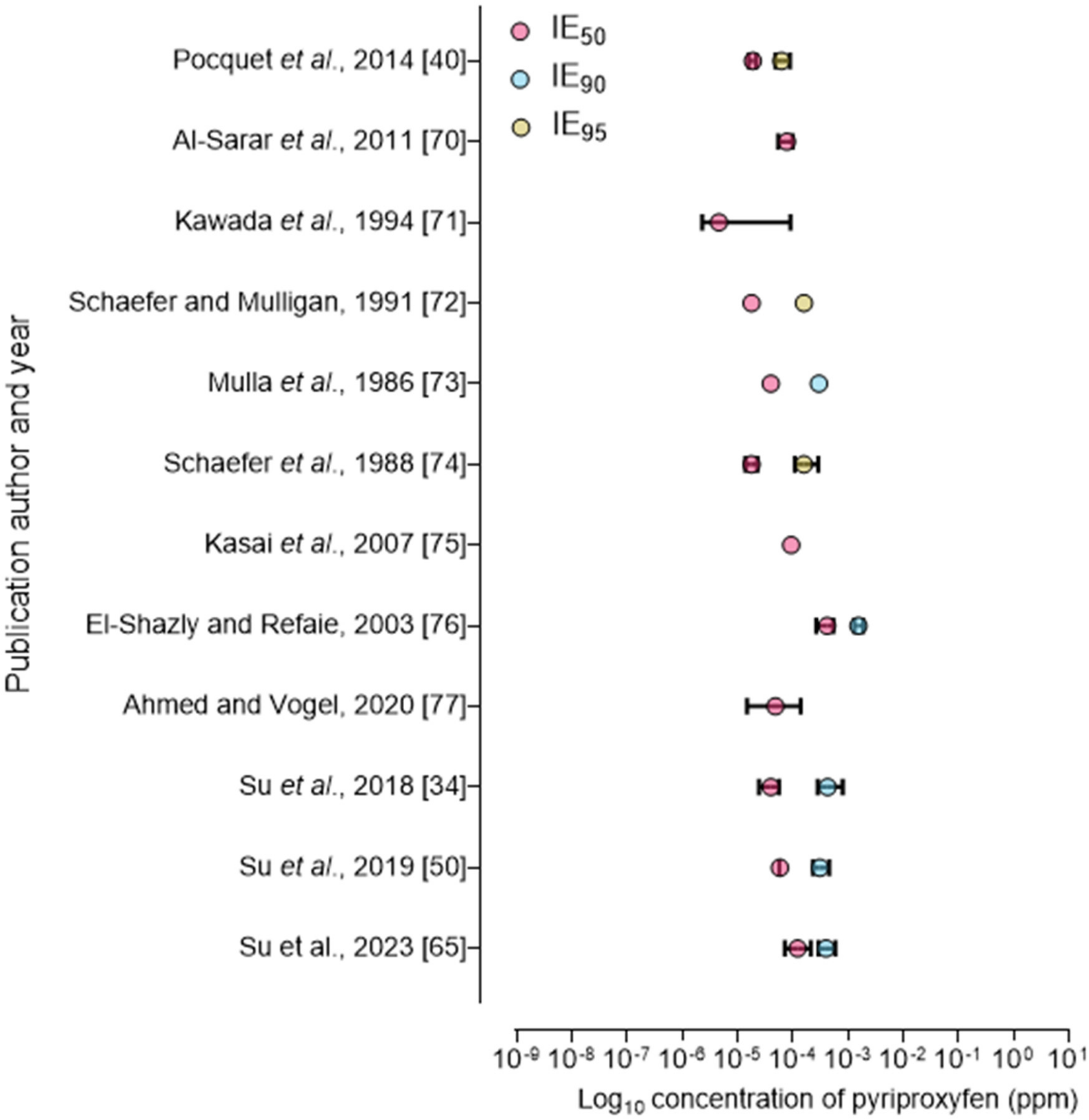

Figure S2. Forest plot of the *Cx. pipiens*/pyriproxyfen values reported in the literature converted to ppm (Log_{10}). IE_{50} values are reported in red (n=12), IE_{90} in blue (n=5) and IE_{95} in yellow (n=3). Confidence intervals (95%) are displayed for publications where uncertainty measurements were reported.

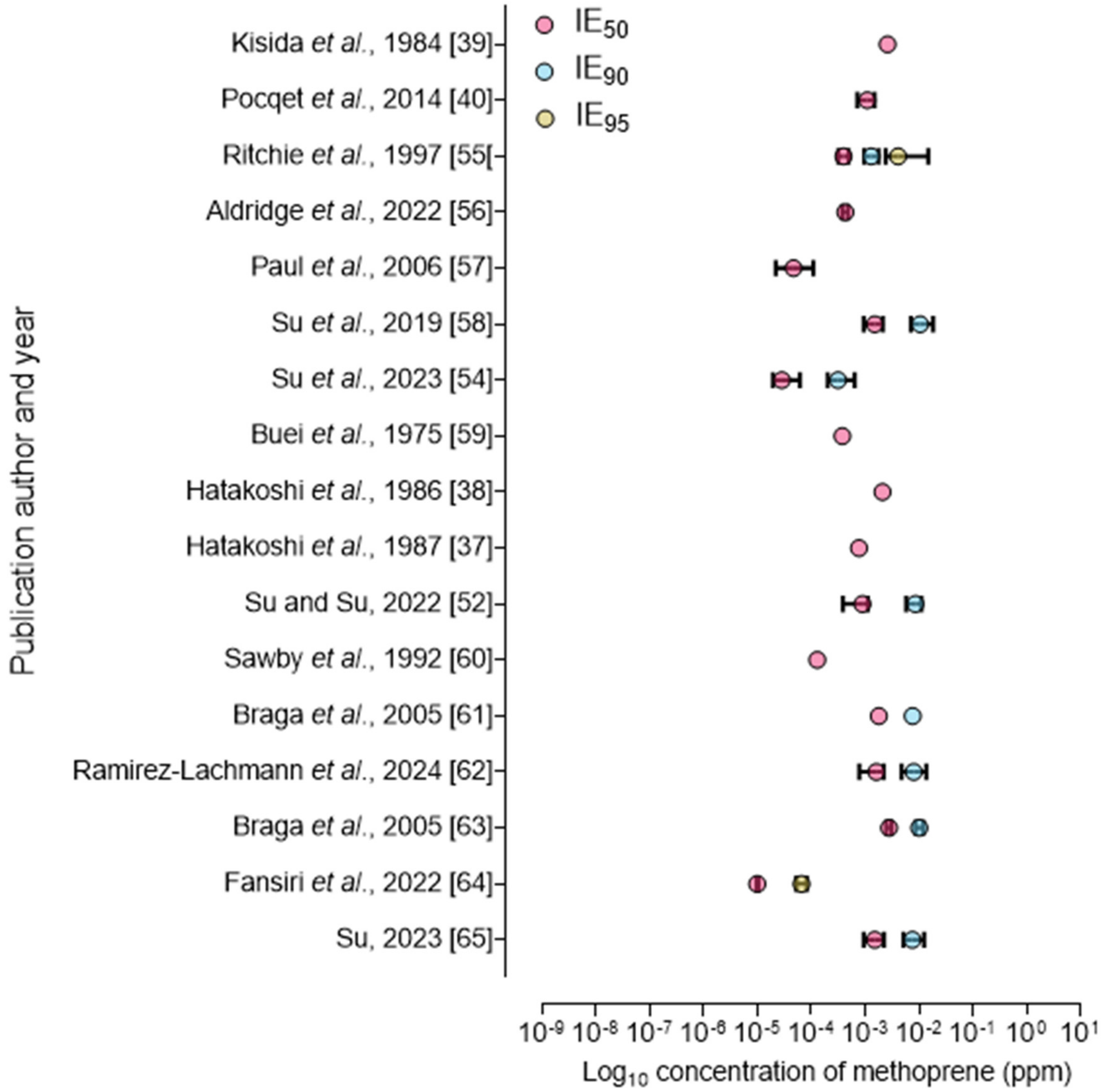

Figure S3. Forest plot of the *Ae. aegypti*/methoprene values reported in the literature converted to ppm (Log_{10}). IE_{50} values are reported in red (n=17) , IE_{90} in blue (n=8) and IE_{95} in yellow (n=2). Confidence intervals (95%) are displayed for publications where uncertainty measurements were reported.

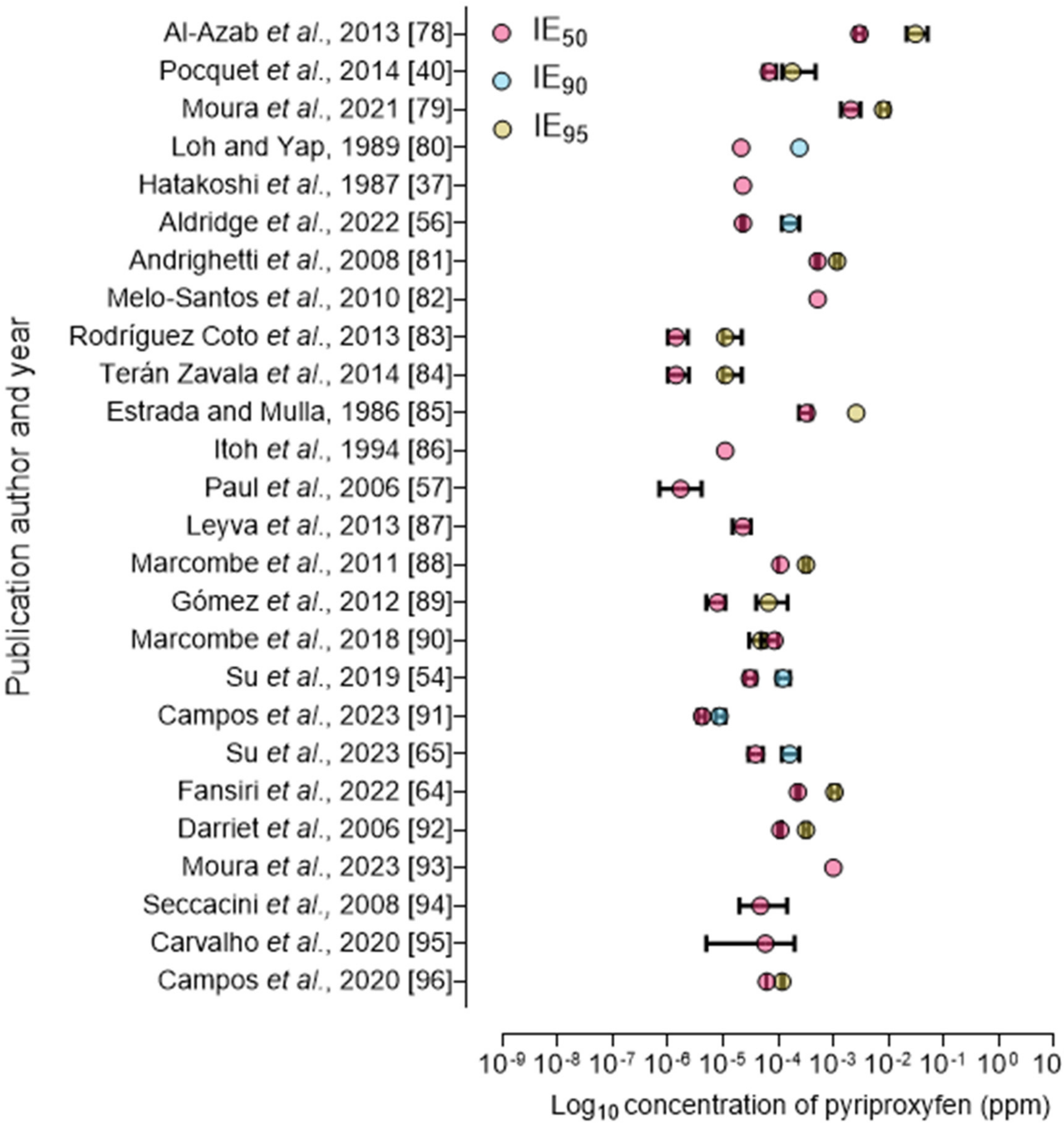

Figure S4. Forest plot of the *Ae. aegypti*/pyriproxyfen values reported in the literature converted to ppm (Log_{10}). IE_{50} values are reported in red (n=26), IE_{90} in blue (n=13) and IE_{95} in yellow (n=5). Confidence intervals (95%) are displayed for publications where uncertainty measurements were reported.

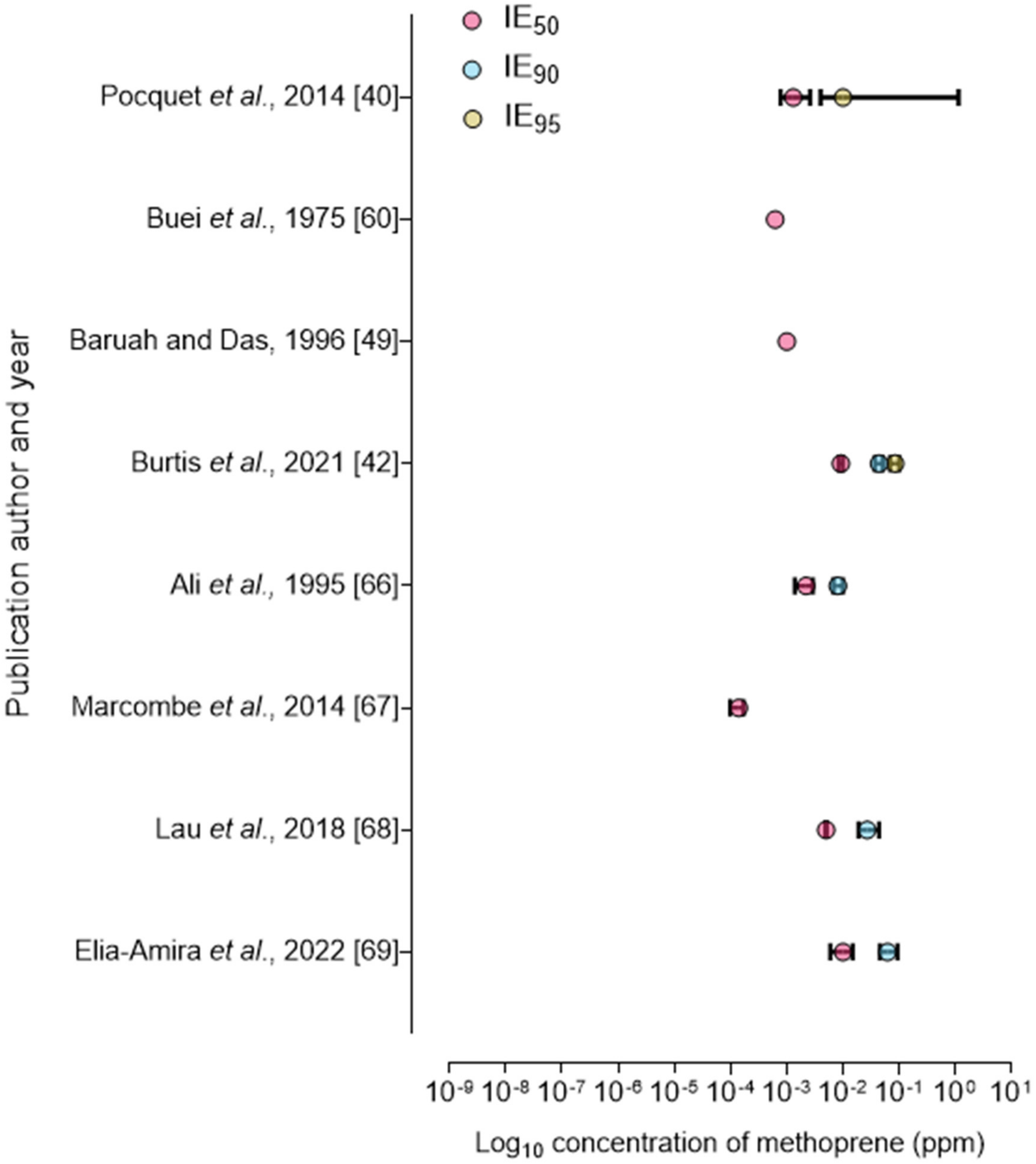

Figure S5. Forest plot of the *Ae. albopictus*/methoprene values reported in the literature converted to ppm (Log_{10}). IE_{50} values are reported in red(n=8), IE_{90} in blue (n=4) and IE_{95} in yellow (n=2). Confidence intervals (95%) are displayed for publications where uncertainty measurements were reported.

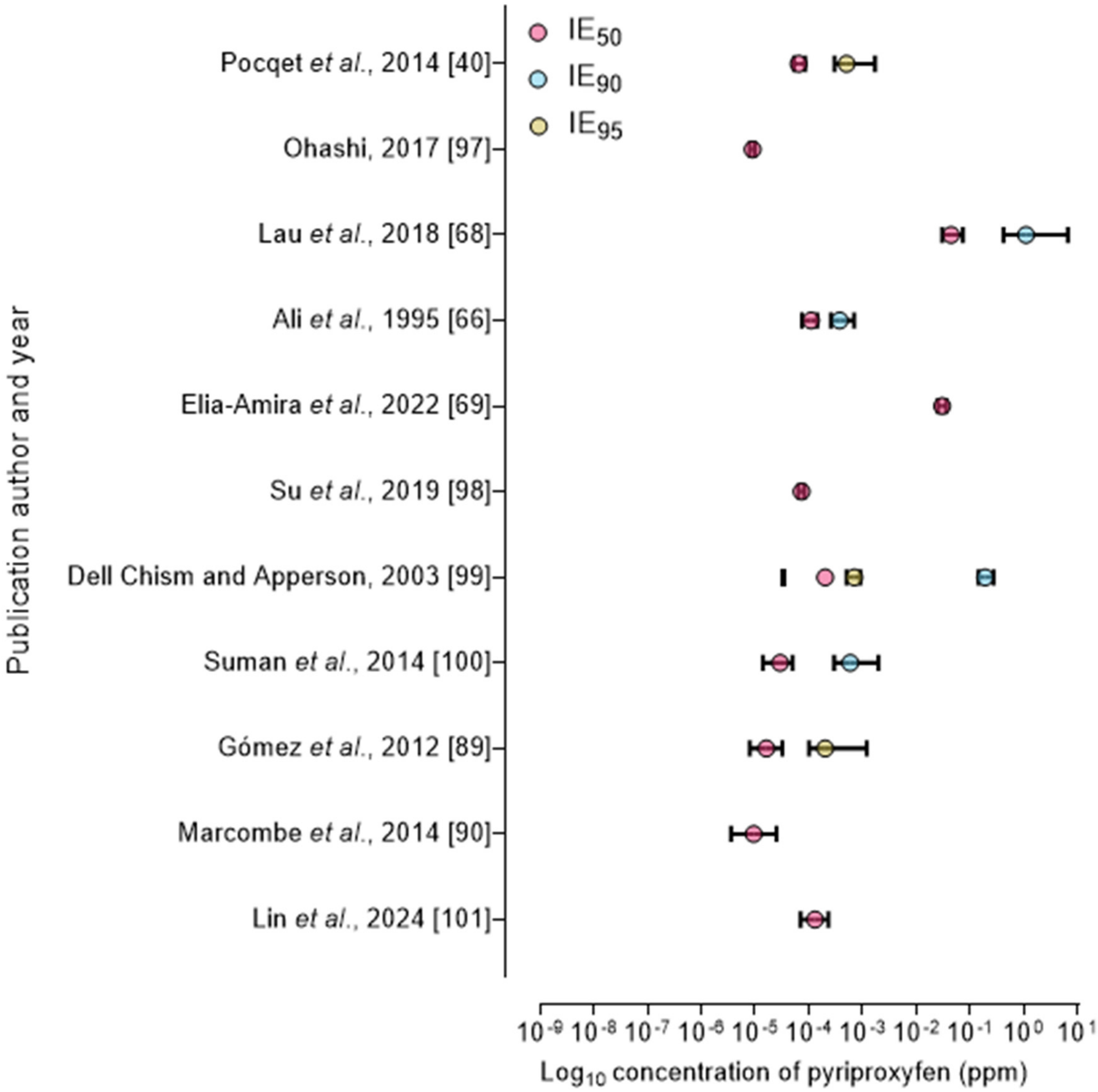

Figure S6. Forest plot of the *Ae. albopictus*/pyriproxyfen values reported in the literature converted to ppm (Log_{10}). IE_{50} values are reported in red (n=11), IE_{90} in blue (n=4) and IE_{95} in yellow (n=3). Confidence intervals (95%) are displayed for publications where uncertainty measurements were reported.

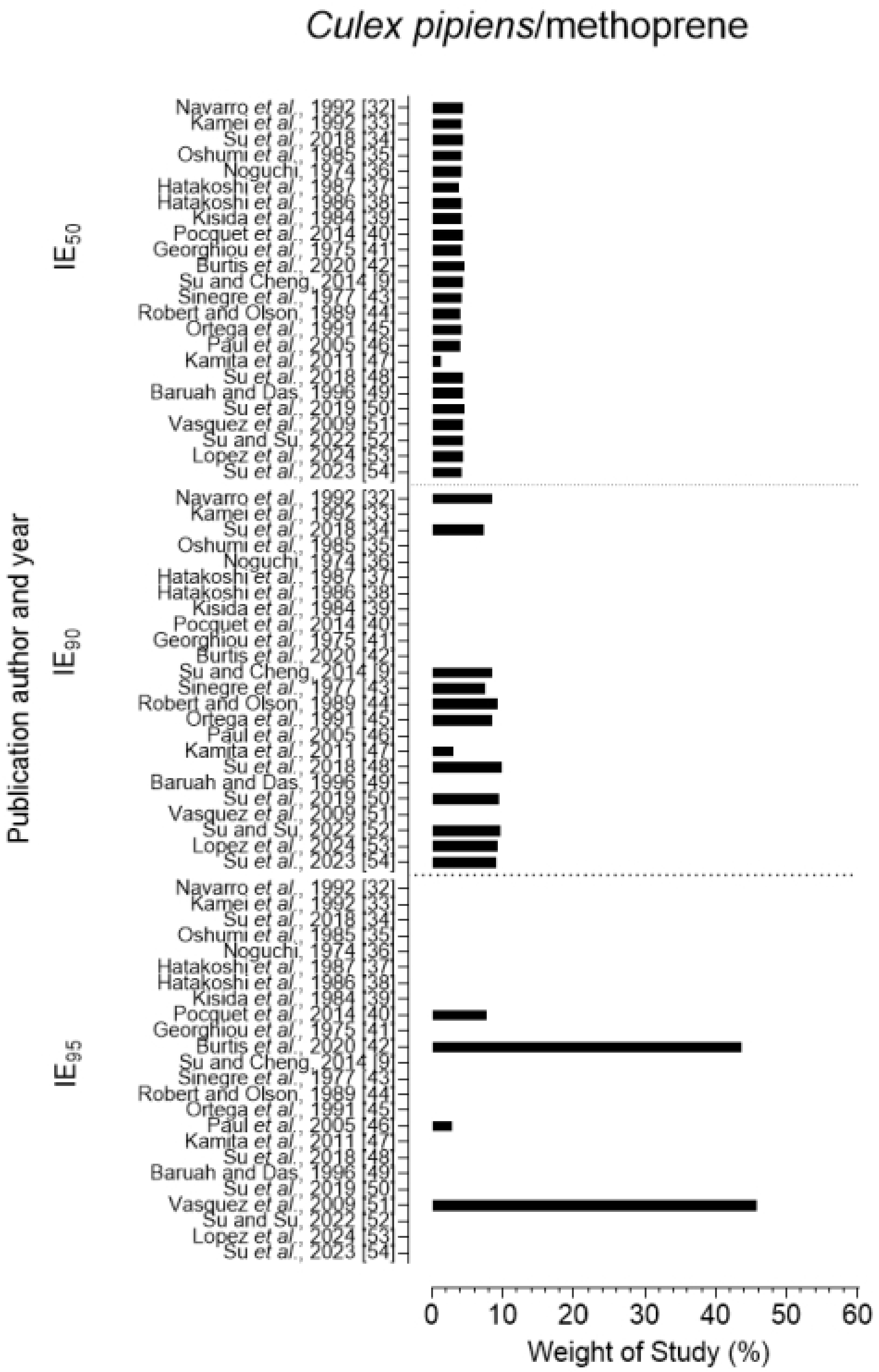

Figure S7. Weighting of each study (in percent) at IE_{50} (n=24), IE_{90} (n=12), and IE_{95} (n=4) in the DerSimonian and Laird inverse weighting random effects model for *Cx. pipiens*/methoprene.

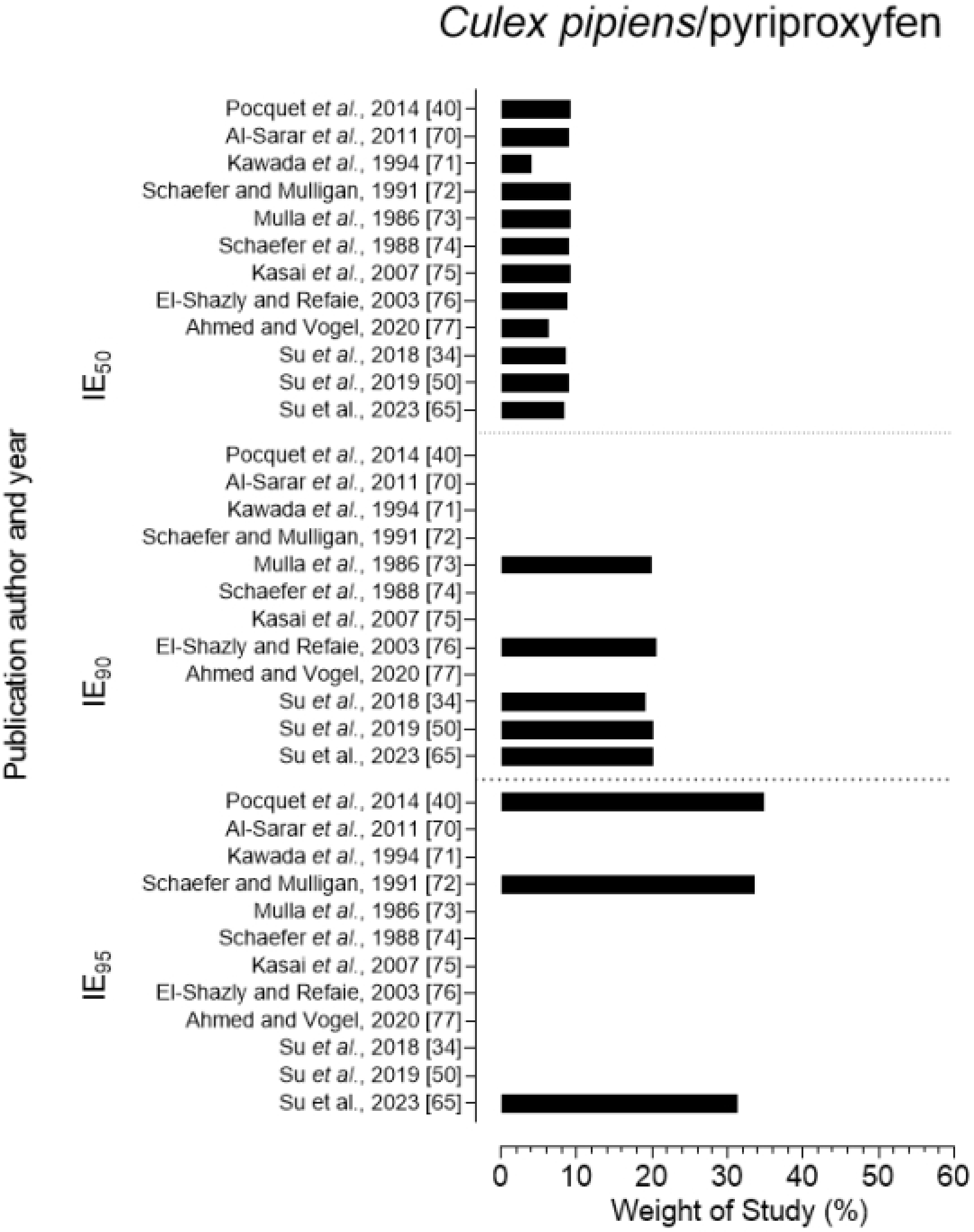

Figure S8. Weighting of each study (in percent) at IE_{50} (n=12), IE_{90} (n=5), and IE_{95} (n=3) in the DerSimonian and Laird inverse weighting random effects model for *Cx. pipiens*/pyriproxyfen.

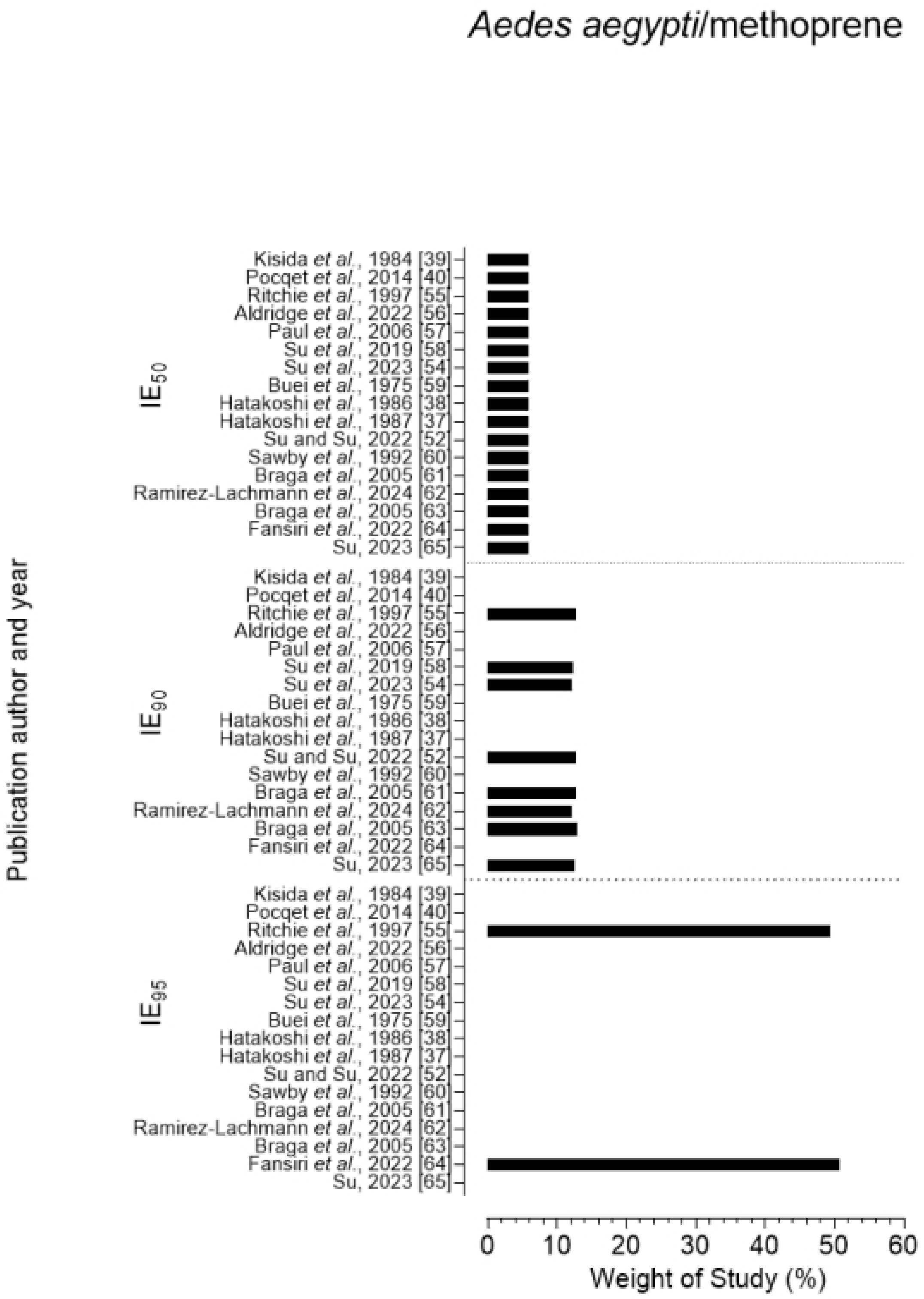

Figure S9. Weighting of each study (in percent) at IE_{50} (n=17), IE_{90} (n=8), and IE_{95} (n=2) in the DerSimonian and Laird inverse weighting random effects model for *Ae. aegypti*/methoprene.

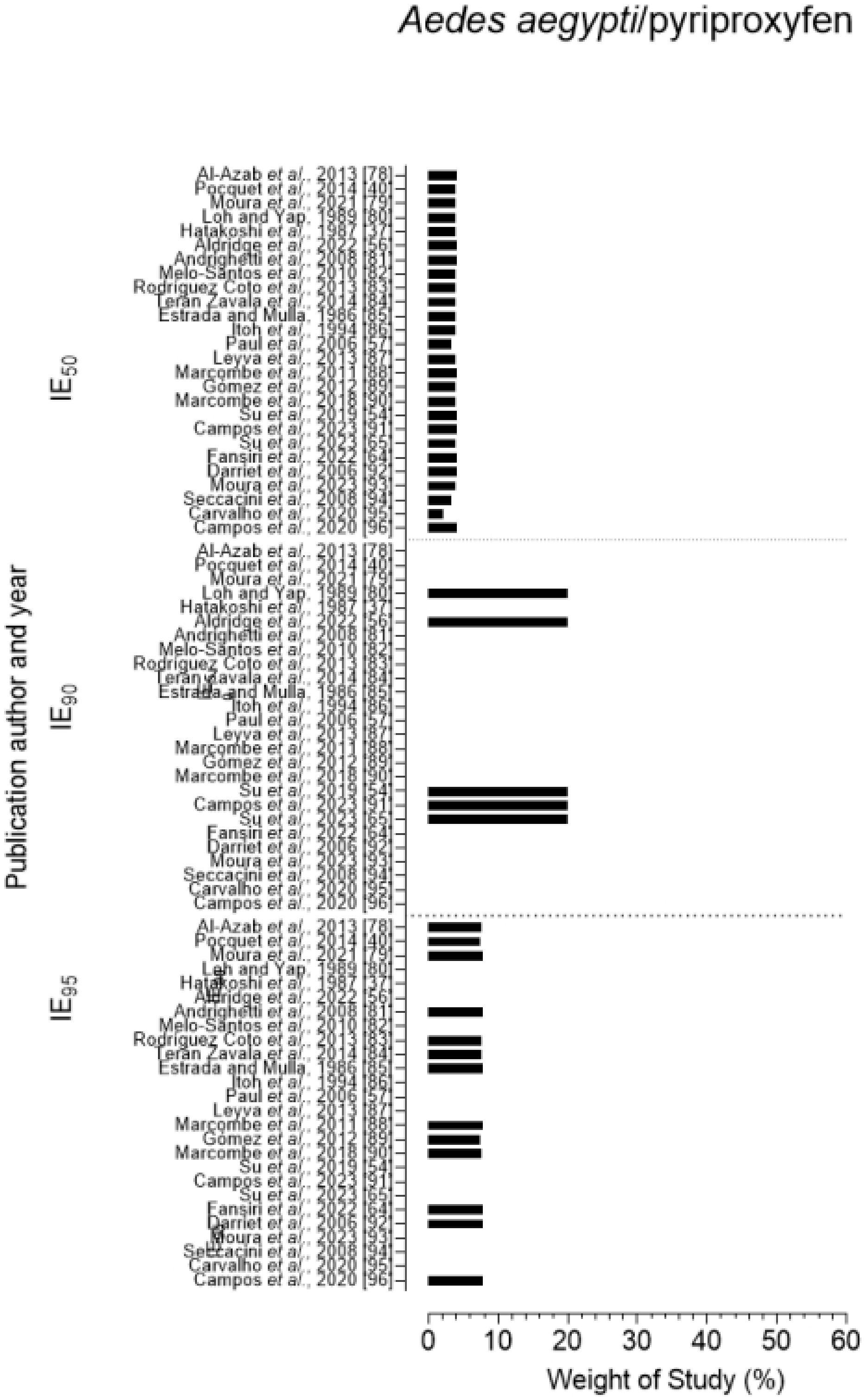

Figure S10. Weighting of each study (in percent) at IE_{50} (n=26), IE_{90} (n=5), and IE_{95} (n=13), in the DerSimonian and Laird inverse weighting random effects model for *Ae. aegypti*/pyriproxyfen.

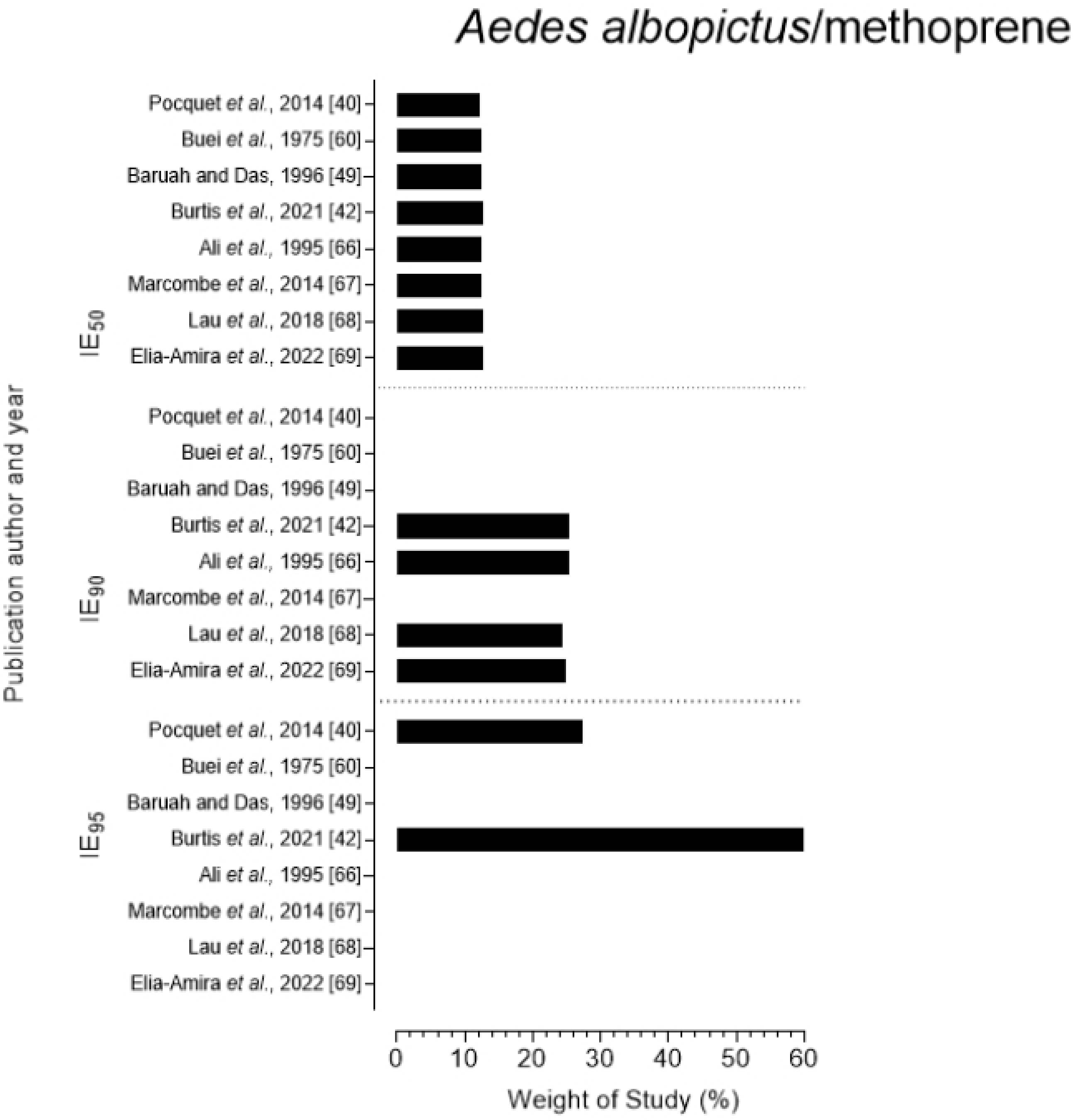

Figure S11. Weighting of each study (in percent) at IE_{50}(n=8), IE_{90}(n=4), and IE_{95} (n=2) in the DerSimonian and Laird inverse weighting random effects model for *Ae. albopictus*/methoprene.

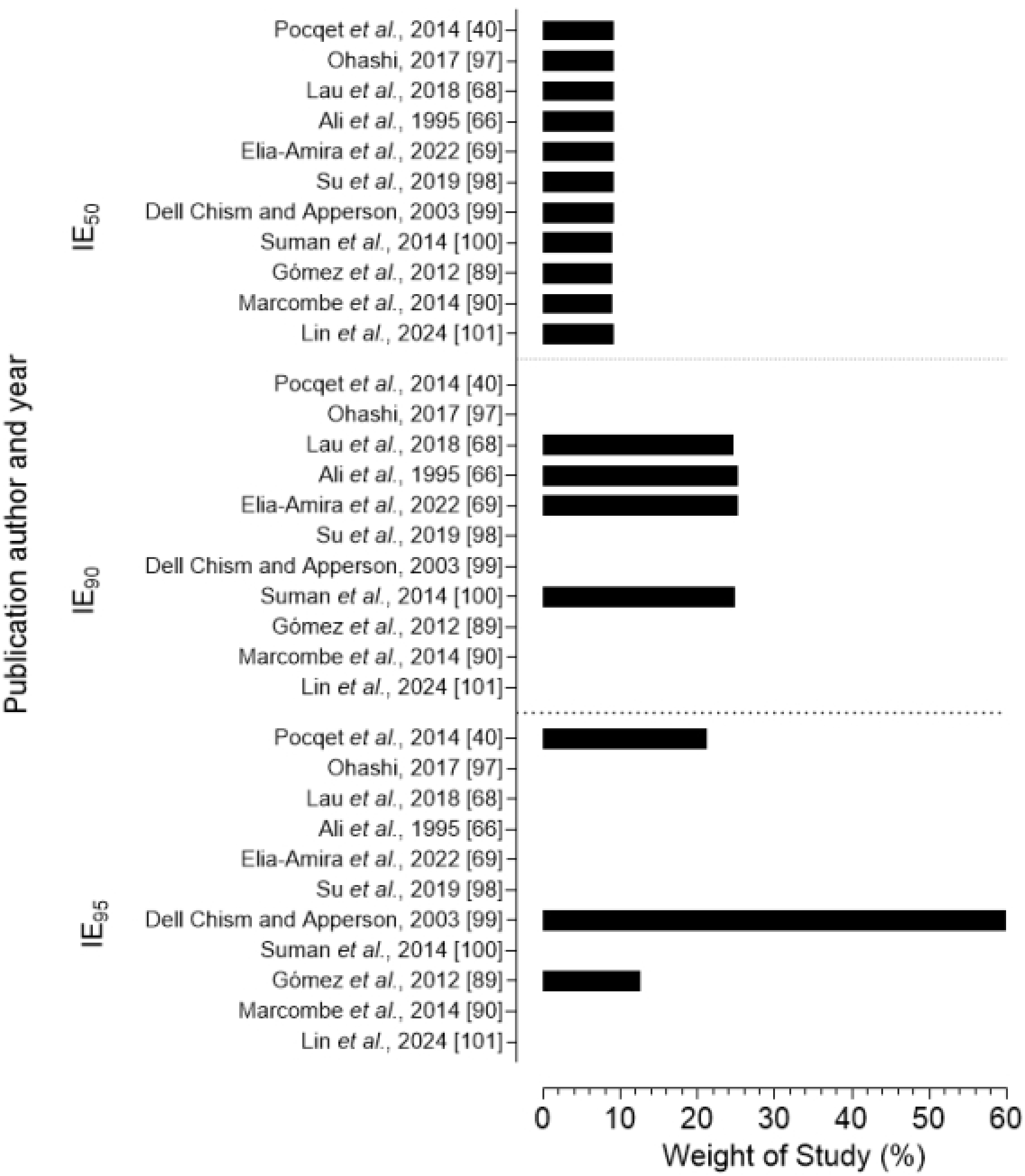

Figure S12. Weighting of each study (in percent) at IE_{50} (n=11), IE_{90} (n=4), and IE_{95}(n=3) in the DerSimonian and Laird inverse weighting random effects model for *Ae. albopictus*/pyriproxyfen.

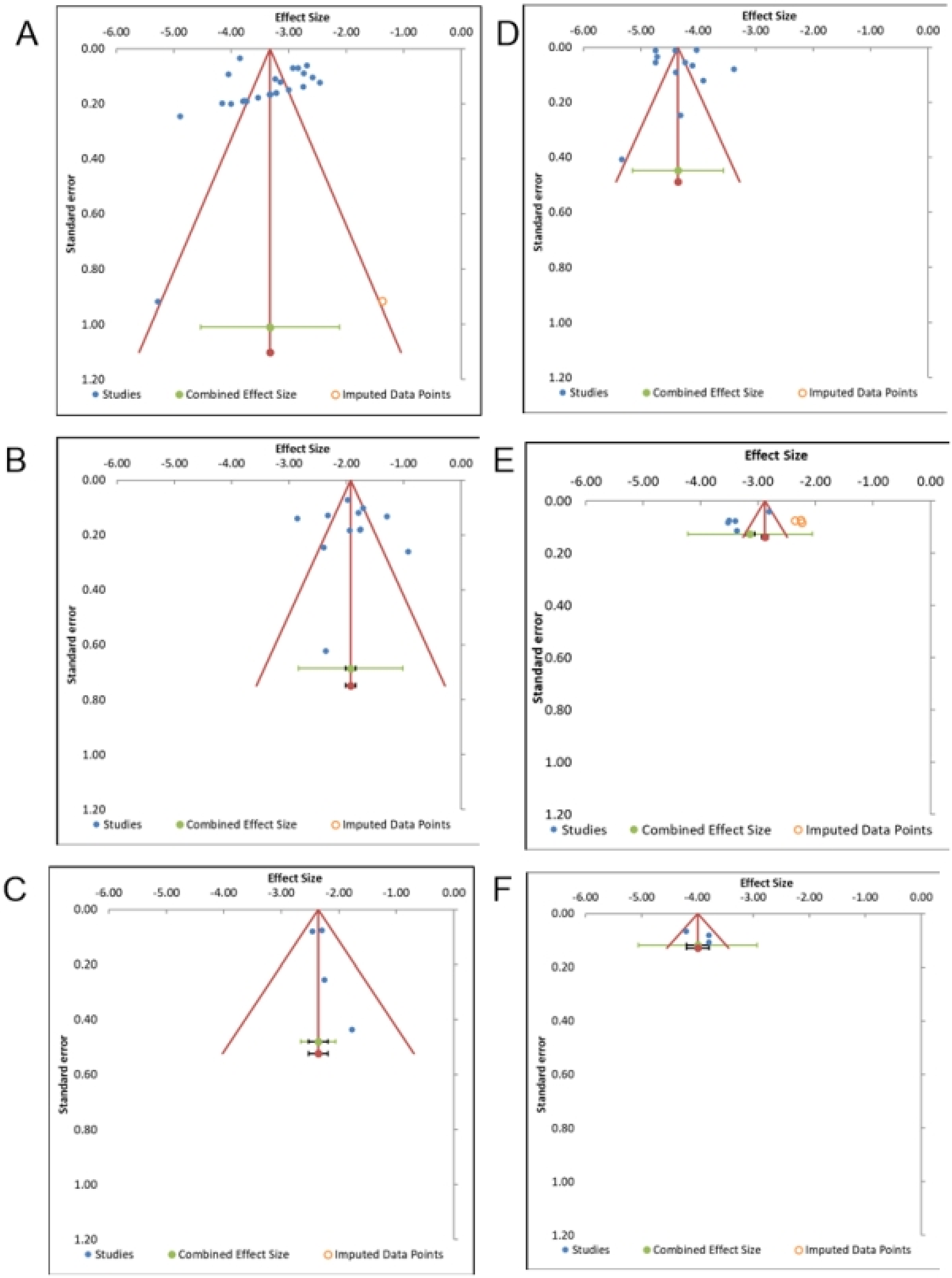

Figure S13. Funnel plot of the published IE values. A) *Cx. pipiens*/methoprene IE_{50}; n=24, B) *Cx. pipiens*/methoprene IE_{90}; n=12, C) *Cx. pipiens*/methoprene IE_{95}; n=4, D) *Cx. pipiens*/pyriproxyfen IE_{50}; n=12, E) *Cx. pipiens*/pyriproxyfen IE_{90}; n= 5, F)*Cx. pipiens*/pyriproxyfen IE_{95}; n=3.

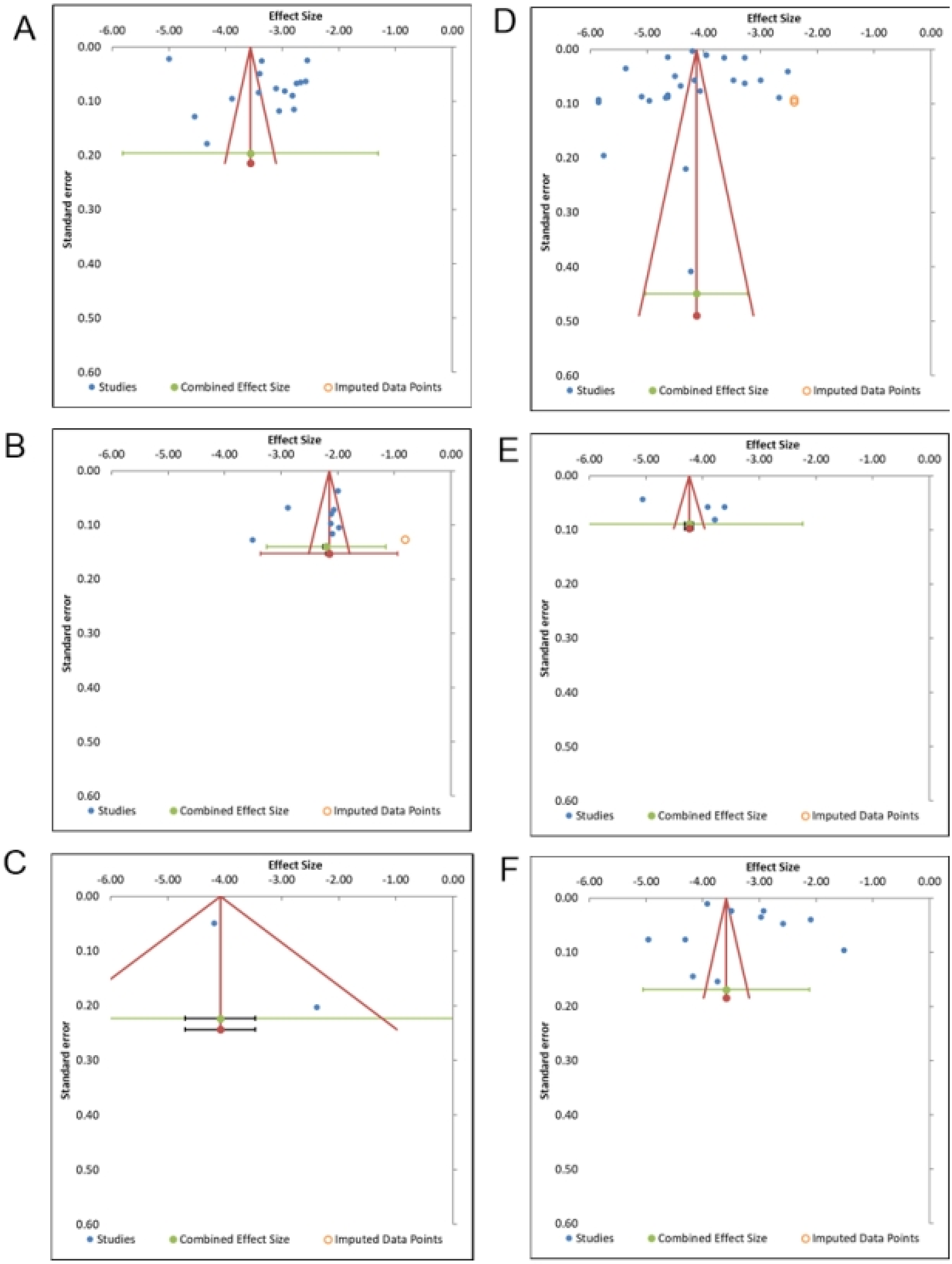

Figure S14. Funnel plot of the published IE values. A) *Ae. aegypti*/methoprene IE$_{50}$; n=17, B) *Ae. aegypti* /methoprene IE$_{90}$; n=8 C) *Ae. aegypti* /methoprene IE$_{95}$; n=2, D) *Ae. aegypti* /pyriproxyfen IE$_{50}$; n=26, E) *Ae. aegypti* /pyriproxyfen IE$_{90}$; n= 13, F) *Ae. aegypti* /pyriproxyfen IE$_{95}$; n=5.

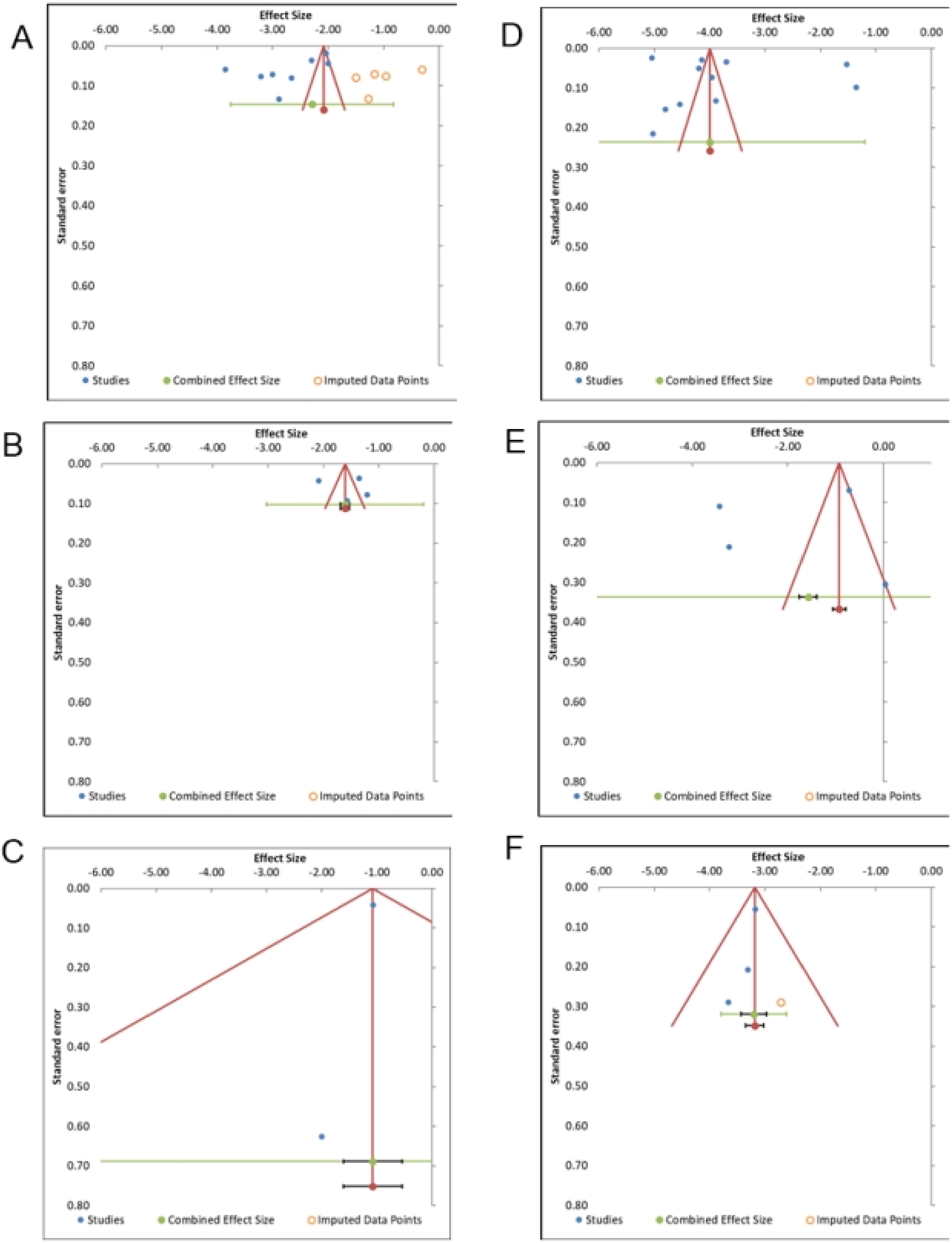

Figure S15. Funnel plot of the published IE values. A) *Ae. albopictus*/methoprene IE_{50}; n=8, B) *Ae. albopictus* /methoprene IE_{90}; n=4 C) *Ae. albopictus* /methoprene IE_{95}; n=2 D) *Ae. albopictus*/pyriproxyfen IE_{50}; n=11 E) *Ae. albopictus* /pyriproxyfen IE_{90}; n=4, F) *Ae. albopictus* /pyriproxyfen IE_{95}; n= 3.
